# Alkyl-Chain Extension and Terminal Amine Substitution Shape Cardiotoxic Profiles of Methylenedioxy Cathinones in Zebrafish Embryos

**DOI:** 10.3390/ph19081243

**Published:** 2026-08-07

**Authors:** Ouwais Aljabasini, Niki Tagkalidou, Martalu D. Pazos, Guillermo García-Díez, Eva Prats, Roger Seco, Xavier Berzosa, Raúl López-Arnau, Demetrio Raldúa

**Affiliations:** 1 Institute of Environmental Assessment and Water Research (IDAEA-CSIC), Jordi Girona, 18, 08034 Barcelona, Spain; ouwais.aljabasini@idaea.csic.es (O.A.); niki.tagkalidou@idaea.csic.es (N.T.); email@rogerseco.cat (R.S.); 2 Department of Pharmacology, Toxicology and Therapeutic Chemistry, Faculty of Pharmacy and Food Sciences, Pharmacology Section, Institute of Biomedicine (IBUB), University of Barcelona, 08028 Barcelona, Spain; martaludominguez@ub.edu (M.D.P.); raullopezarnau@ub.edu (R.L.-A.); 3 Chemical Reactions for Innovative Solutions (CRISOL), IQS School of Engineering, Universitat Ramon Llull, 08017 Barcelona, Spain; guillermo.garcia@iqs.url.edu (G.G.-D.); xavier.berzosa@iqs.url.edu (X.B.); 4 Research and Development Center (CID-CSIC), Jordi Girona, 18, 08034 Barcelona, Spain; eva.prats@cid.csic.es

**Keywords:** synthetic cathinones, new psychoactive substances, 3,4-methylenedioxy cathinones, methylone, pentylone, dipentylone, zebrafish embryo, cardiotoxicity, atrioventricular block, structure-activity relationship, New Approach Methodologies

## Abstract

**Background/Objectives**: Synthetic cathinones are a rapidly evolving class of new psychoactive substances whose structural diversity complicates toxicological risk assessment. Methylenedioxy cathinones occupy a pharmacological space between MDMA-like entactogens and more dopaminergic stimulant cathinones, but their direct cardiac liabilities remain poorly characterized. This study aimed to compare the cardiotoxic and neurobehavioral profiles of methylone, butylone, pentylone and their N,N-dimethyl analogues, and to determine how alkyl-chain extension and terminal amine substitution shape functional toxicity. **Methods**: Wild-type short-fin zebrafish (*Danio rerio*) embryos were used as a multiparametric New Approach Methodology. Cardiac rhythmicity was assessed in 3 days post-fertilization embryos after acute exposure to methylone, butylone, pentylone, dimethylone, dibutylone, dipentylone, dihexylone and diheptylone by high-speed video microscopy and dynamic pixel-based analysis, focusing on atrial chronotropy and atrioventricular conduction. Basal locomotor activity was evaluated in 5 days post-fertilization eleutheroembryos over 120 min using automated video tracking. **Results**: Negative chronotropy increased with alkyl-chain extension, with the monoalkyl subset following the rank order methylone < butylone < pentylone. Among dialkyl analogues, dihexylone and, especially, diheptylone produced the strongest atrial-rate inhibition. AV conduction impairment was more heterogeneous but became prominent among higher-liability analogues, with diheptylone showing the lowest AV-block midpoint descriptor and complete lethality at 1000 µM. Locomotor profiling revealed predominantly hypoactive phenotypes, with sustained late-phase inhibition especially for dipentylone, dihexylone and diheptylone. **Conclusions**: Alkyl-chain extension and terminal amine substitution shaped cardiac and neurobehavioral toxicity in a structure-dependent manner. The zebrafish workflow provides a structure-oriented framework for prioritizing emerging methylenedioxy cathinones with comparatively higher functional cardiac liability.

## 1. Introduction

New psychoactive substances (NPS) remain a major challenge for public health, clinical toxicology and forensic surveillance because minor chemical modifications can generate new analogues with uncertain pharmacological and toxicological profiles [1,2,3]. Synthetic cathinones constitute one of the most prominent stimulant subclasses of NPS. These compounds are beta-keto analogues of amphetamine-like phenethylamines derived from cathinone, the psychoactive alkaloid of *Catha edulis*, and they are commonly designed to reproduce stimulant, empathogenic and/or rewarding and reinforcing effects associated with amphetamine, cocaine or MDMA-like drugs [1,2,3,4].

The primary pharmacology of synthetic cathinones is mediated by monoamine transporters, principally the dopamine transporter (DAT), norepinephrine transporter (NET) and serotonin transporter (SERT) [2,3,4,5]. Depending on their structural features, cathinones can behave as cocaine-like uptake inhibitors, amphetamine-like transporter substrates/releasers, or hybrid molecules combining transporter blockade and substrate activity [2,5,6,7]. Structure-activity relationship (SAR) studies have shown that substitutions on the aromatic ring, variation in the alpha-alkyl side chain, and modification of the terminal amine strongly influence transporter potency, DAT/SERT selectivity, psychostimulant efficacy, abuse liability, and toxicity [3,6,7,8,9].

**Figure 1:**
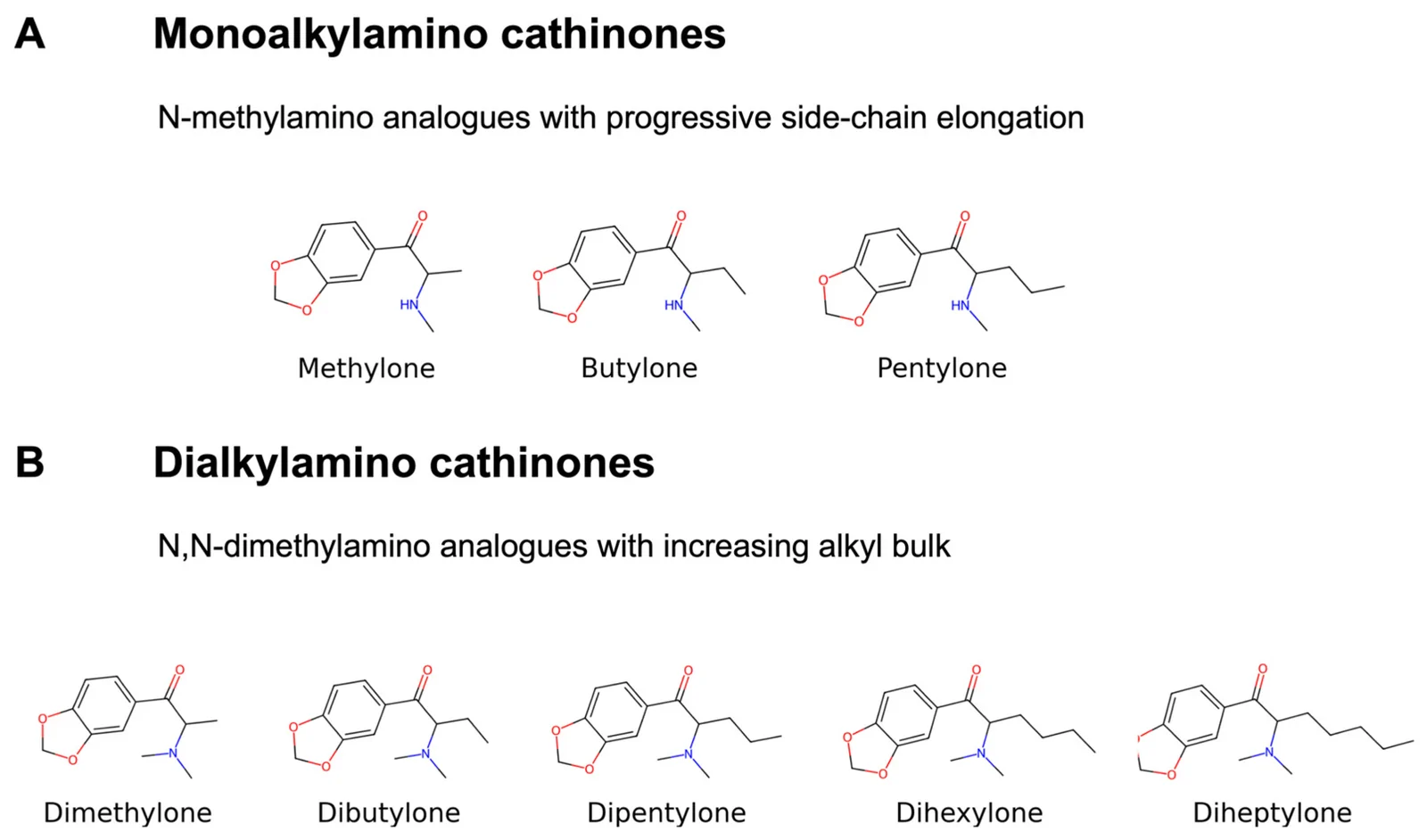
Chemical structures of the tested methylenedioxy cathinones and qualitative SAR organization.

Methylenedioxy cathinones represent a structurally coherent subgroup characterized by a 1,3-benzodioxol-5-yl aromatic ring and a non-cyclic aminoalkyl side chain. Methylone, butylone and pentylone are N-methyl analogues that differ primarily in alpha-alkyl chain length, whereas dimethylone, dibutylone, dipentylone and longer dialkyl analogues contain a tertiary N,N-dimethylamino group. This homologous organization makes the group especially suitable for SAR analysis, because two main determinants can be compared: progressive alpha-alkyl chain elongation and terminal amine substitution. The chemical structures and qualitative SAR organization of the tested compounds are shown in Figure 1.

Several methylenedioxy cathinones have been pharmacologically characterized. Methylone acts as a monoamine transporter substrate with an MDMA-like profile [4]. Butylone and pentylone have been described as stimulants that block DAT while retaining SERT substrate activity, with pentylone generally showing greater dopaminergic selectivity than butylone [5]. In vivo studies in rodents further show that methylone, pentylone and related cathinones increase locomotor activity and display reinforcing and/or discriminative-stimulus effects, with structural differences influencing the balance between MDMA-like and stimulant-like profiles [6,7]. A broader SAR analysis of second-generation cathinones has also shown that N-terminal substitution and ring substitution can modify DAT/SERT selectivity, psychostimulant effects, and reward-related molecular markers [8].

The N,N-dimethyl-cathinone analogues have gained additional scientific interest and forensic relevance. For instance, dimethylone and dibutylone have been evaluated in rodent behavioural assays and shown to produce stimulant-like effects [9]. Importantly, N,N-dimethylpentylone, also known as dipentylone or dimethylpentylone, has been identified in postmortem casework, seized samples and counterfeit ecstasy or molly-type drug supplies [10,11]. These observations indicate that the tertiary-amine members of this series are not only theoretical SAR comparators but also relevant emerging compounds in the illicit stimulant market which may pose a public health threat.

Cardiovascular toxicity is a recurrent concern in synthetic cathinone intoxication. Clinical and forensic reports have associated synthetic cathinones with tachycardia, hypertension, hyperthermia, chest pain, myocardial injury, cardiac arrest and arrhythmias [1,12,13]. However, human case interpretation is often complicated by uncertain dose, adulteration, polydrug exposure, and limited analytical confirmation. Consequently, direct experimental data are needed to determine whether structural modifications that increase psychostimulant potency also increase cardiac liability, or whether neurobehavioral and cardiac domains can become dissociated across closely related analogues.

Zebrafish embryos provide a useful New Approach Methodology (NAM) for this question because they allow rapid, non-invasive and multiparametric functional assessment in vivo [14]. The transparency of embryos enables direct imaging of cardiac function, while their small size and external development support medium-throughput screening. Importantly, zebrafish express an hERG/KCNH2 orthologue and are sensitive to drugs that induce repolarization abnormalities, bradycardia, and atrioventricular conduction phenotypes, supporting their utility for functional cardiotoxicity screening [15,16,17]. In parallel, at 5 dpf, zebrafish eleutheroembryos display robust spontaneous locomotor activity and pharmacologically responsive behavioural outputs, making them suitable for detecting drug-induced alterations in neurobehavioral function [18,19]. In the NPS field, first-generation cathinones, including methylone and MDPV, have been shown to produce arrhythmia-related phenotypes in zebrafish eleutheroembryos [20]. Our previous integrated study with pyrrolidine-containing cathinones further demonstrated that subtle structural modifications can produce distinct patterns of atrial chronotropy, AV conduction impairment and locomotor disruption [21].

In the present study, we evaluated the cardiotoxic and neurobehavioral effects of a panel of methylenedioxy cathinones spanning N-methyl and N,N-dimethyl analogues: methylone, butylone, pentylone, dimethylone, dibutylone, dipentylone, dihexylone and diheptylone. We hypothesized that alpha-alkyl chain extension and terminal amine substitution would shape cardiac rhythmicity, AV conduction and locomotor output in a structure-dependent manner. By combining concentration-response modelling of atrial chronotropy and AV block with time-resolved locomotor profiling, this work provides a comparative SAR framework for prioritizing emerging methylenedioxy cathinones with increased functional cardiac liability and for determining whether cardiac and neurofunctional effects scale in parallel across this homologous series.

## 2. Results

### 2.1. Effects on Atrial Chronotropy

All tested compounds altered cardiac function in zebrafish embryos, but the intensity of the response differed substantially across the series. Atrial rate inhibition increased with concentration for all compounds except methylone, which remained comparatively weak across the tested range. Across both the monoalkyl and dialkyl subsets, progressive alkyl chain elongation was generally associated with stronger negative chronotropy.

Within the monoalkyl subgroup, mean atrial inhibition increased from 0.3%, 23.4% and 32.9% for methylone to 16.2%, 34.4% and 58.1% for butylone, and to 42.1%, 55.1% and 77.3% for pentylone at 250, 500 and 1000 µM, respectively. This pattern supports a rank order of methylone < butylone < pentylone for negative chronotropic activity.

A related trend was observed among the dialkyl analogues. Dimethylone and dibutylone induced modest-to-moderate atrial inhibition, whereas dipentylone and dihexylone produced stronger effects. Diheptylone showed the most severe atrial depression among the dialkyl analogues at the non-lethal concentrations tested, reaching mean inhibitions of 60.4% and 69.1% at 250 and 500 µM, respectively. However, this interpretation requires caution because partial mortality occurred for diheptylone at 500 µM, and complete lethality occurred at 1000 µM. Partial mortality was also observed for dihexylone at 1000 µM. Functional cardiac values at diheptylone 500 µM and dihexylone 1000 µM were therefore calculated only from surviving embryos and should be interpreted together with the mortality/exclusion data reported in Table 1. The diheptylone 1000 µM condition was excluded from curve fitting and interpreted as overt embryotoxicity rather than as a conventional cardiac endpoint. The experimental design, replication structure, exclusion criteria and recording conditions are summarized in Supplementary Table S1, and the statistical approach used for cardiac endpoint analysis is summarized in Supplementary Table S2. The full Supplementary Dataset package is organized as Supplementary Datasets S1–S6, comprising cardiac chamber heart-rate data, cardiac endpoint statistics, normalized BLA/log_2_ fold-change values, raw immobility parameters, time-resolved normalized distance/GEE results and experimental metadata/exclusion definitions, respectively.

**Figure 2:**
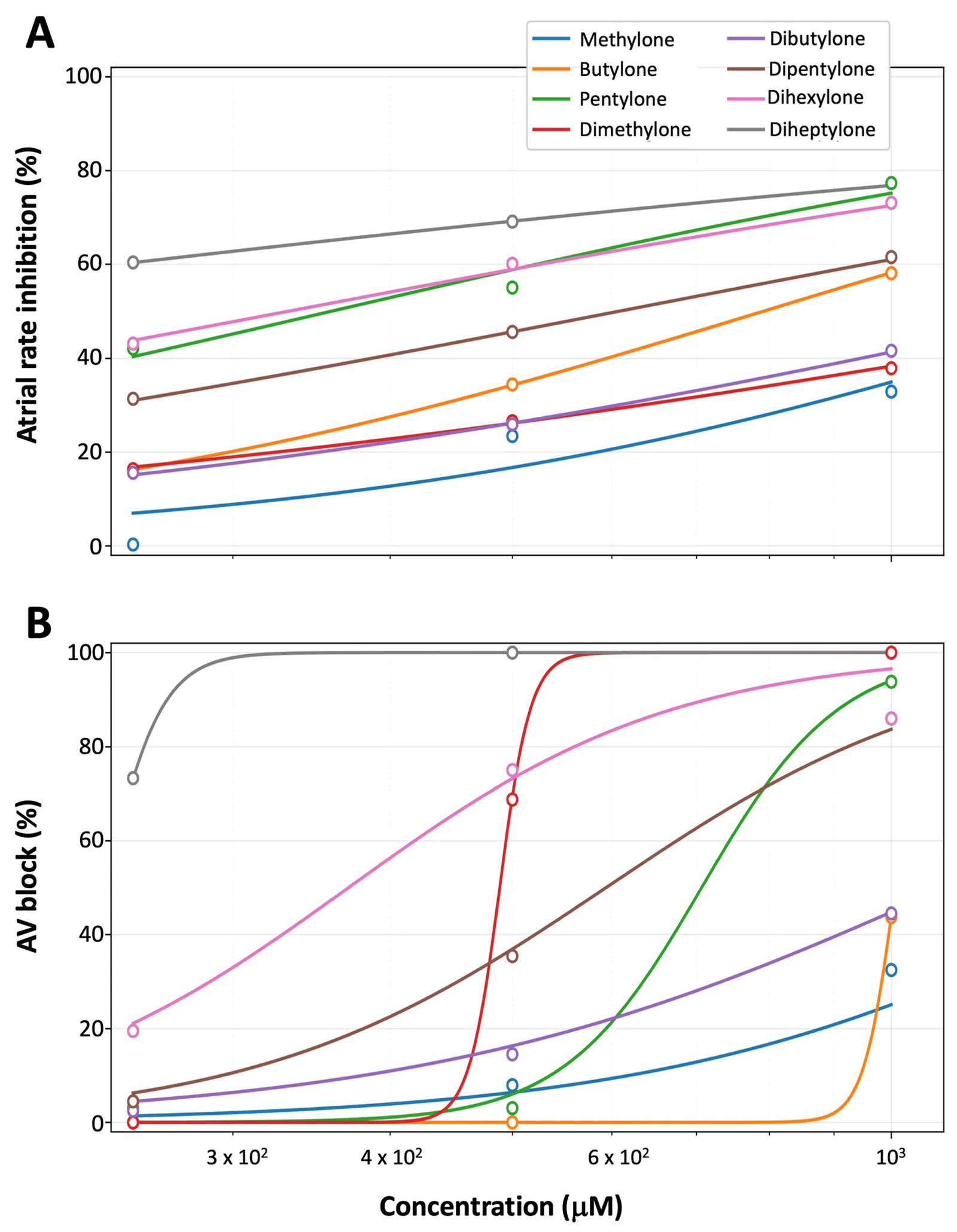
Concentration-response profiles for cardiotoxicity across the methylenedioxy cathinone series. (**A**) Atrial rate inhibition; (**B**) atrioventricular (AV) conduction impairment. Open circles indicate observed mean responses at the tested concentrations, and lines indicate constrained 4PL fits.

Constrained 4PL-derived atrial chronotropy descriptors are summarized in Table 2. Diheptylone had the lowest atrial EC_50_ estimate (118.3 µM), followed by dihexylone (332.5 µM) and pentylone (359.6 µM). Methylone, dimethylone and dibutylone were among the least potent negative chronotropic compounds in the series. The corresponding atrial concentration-response profiles are shown in Figure 2A.

### 2.2. Effects on Atrioventricular Conduction

AV conduction disturbances also increased with exposure concentration, but the relationship with chemical structure was more heterogeneous than for atrial rate. The shorter and less toxic analogues mainly produced rate depression with limited AV block, whereas the higher-liability members of the series increasingly induced conduction defects, particularly a 2:1 AV block phenotype.

Within the monoalkyl series, methylone produced little AV block at 250 and 500 µM, with a clearer effect only at 1000 µM. Butylone also showed minimal AV impairment at lower concentrations but reached 43.8% AV block at 1000 µM. Pentylone was more disruptive, culminating in 93.8% AV block at 1000 µM. These data suggest that chain elongation in the monoalkyl subset enhances not only negative chronotropy but also susceptibility to atrial-ventricular uncoupling.

Among the dialkyl derivatives, AV liability became more prominent. Dimethylone produced 68.8% AV block at 500 µM and 100.0% at 1000 µM, while dipentylone and dihexylone displayed strong conduction phenotypes at the upper concentrations. Diheptylone was the most severe compound in this endpoint as well, already producing 73.3% AV block at 250 µM and complete AV block among surviving embryos at 500 µM. However, this 500 µM value should be interpreted together with the partial mortality observed at the same concentration, because functional AV measurements necessarily included only embryos that remained viable at the time of recording. Partial mortality was also observed for dihexylone at 1000 µM. As with the atrial endpoint, the 1000 µM diheptylone condition was excluded from functional analysis because no viable embryos remained and was interpreted as overt embryotoxicity rather than as a conventional AV conduction endpoint.

Constrained 4PL-derived AV-block descriptors are summarized in Table 3. Diheptylone showed the lowest AV block EC50 estimate (237.3 µM), followed by dihexylone (370.8 µM), dimethylone (488.8 µM), dipentylone (593.7 µM) and pentylone (707.1 µM). Methylone and dibutylone displayed weaker or less stable concentration-response estimates, consistent with the broader or non-estimable confidence intervals observed in these compounds. The corresponding AV conduction concentration-response profiles are shown in Figure 2B. The normalized atrial and ventricular heart-rate values underlying the cardiac analyses are provided in Supplementary Dataset S1. Representative cardiac recordings illustrating normal 1:1 atrioventricular coupling, severe negative chronotropy with preserved 1:1 coupling, and 2:1 atrioventricular conduction phenotypes in high-liability compounds are provided as Supplementary Videos S1–S5.

### 2.3. Diheptylone Showed the Highest Overall Cardiac Liability

Diheptylone consistently emerged as the analogue with the highest overall cardiac liability in the current dataset. Unlike the rest of the series, it produced pronounced atrial depression and AV conduction failure already at the lowest tested concentration. This leftward functional shift was accompanied by partial mortality at 500 µM and complete lethality at 1000 µM, indicating that the toxic response exceeded a purely electrophysiological phenotype and progressed to overt embryotoxicity. Partial mortality was also observed for dihexylone at 1000 µM, indicating that the two longest-chain analogues approached or exceeded the range in which functional cardiac endpoints can be interpreted independently from overt embryotoxicity.

From an interpretation standpoint, the diheptylone profile is important because it sets an upper limit to the apparent SAR trend: further alkyl extension was associated not only with stronger functional cardiac effects but also with a transition toward non-specific lethality within the tested range.

### 2.4. Effects on Locomotor Activity

**Figure 3:**
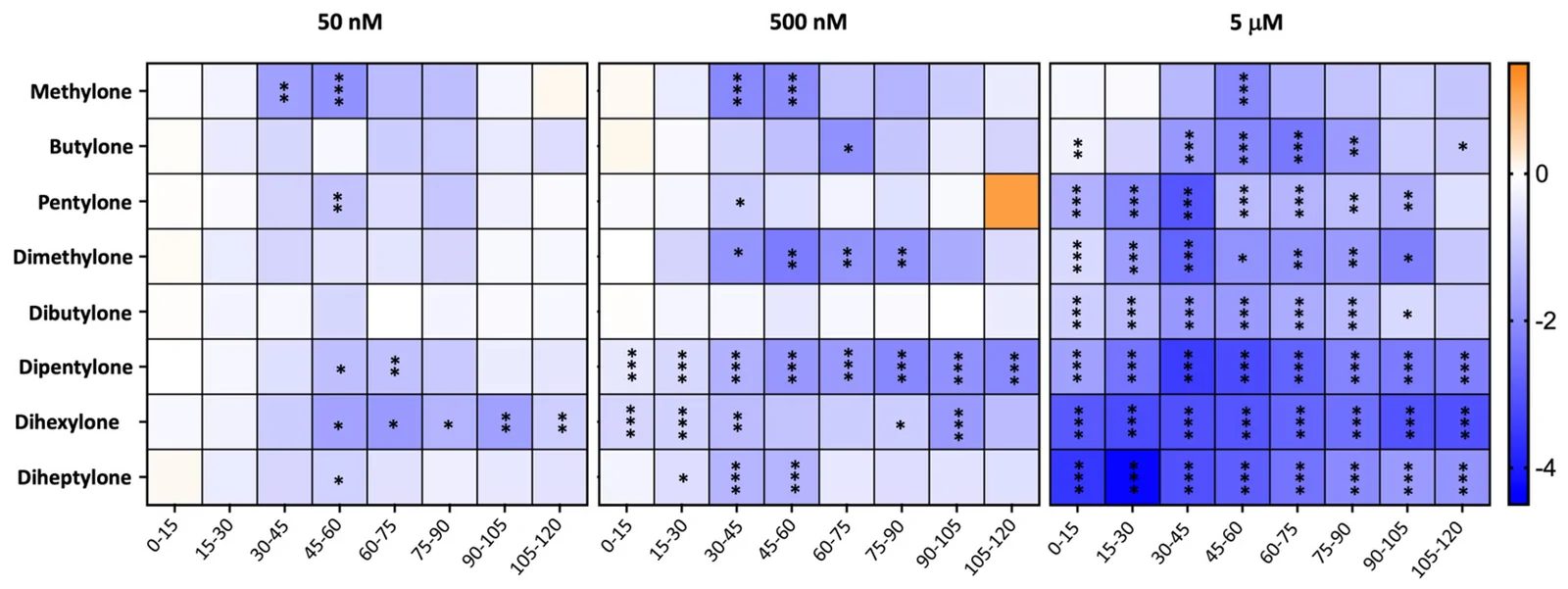
Time-resolved effects of methylenedioxy cathinones on zebrafish eleutheroembryos’ locomotor activity. Heatmaps show log_2_ fold-change values in locomotor activity relative to the corresponding control group across consecutive 15 min recording intervals. Eleutheroembryos were exposed to each compound at 50 nM, 500 nM, or 5 µM. Rows indicate the tested compounds and columns indicate the recording intervals from 0 to 120 min. Negative values indicate reduced locomotor activity relative to controls, whereas positive values indicate increased activity. The same colour scale was applied to all concentrations. Statistical significance was assessed independently for each concentration and recording interval using the Kruskal–Wallis test followed by post hoc multiple comparisons against the corresponding control group. Asterisks indicate significant differences versus the corresponding control group: * *p* < 0.05, ** *p* < 0.01, and *** *p* < 0.001. Time-resolved distance-travelled values normalized to the corresponding time-matched control median are shown in Supplementary Figure S1, and the corresponding descriptive statistics and repeated-measures GEE results are provided in Supplementary Dataset S5.

Time-resolved basal locomotor activity (BLA) was assessed in 5 dpf zebrafish eleutheroembryos during continuous exposure to 50 nM, 500 nM and 5 µM of each compound over 120 min, using consecutive 15 min intervals (Figure 3). Overall, the series produced compound-, concentration- and time-dependent changes in locomotor output. The dominant phenotype was hypoactivity rather than sustained hyperlocomotion, although the magnitude and persistence of the effect differed markedly across analogues. The median normalized BLA values and corresponding log2 fold-change matrix used for Figure 3 are provided in Supplementary Dataset S3. Late-phase descriptive statistics for normalized BLA and complete representative-window nonparametric statistics are provided in Supplementary Tables S3 and S4, respectively.

To complement the normalized heatmap, time-resolved distance-travelled values were also summarized as a percentage of the corresponding time-matched control median. This normalization was used to account for variability between independent experimental runs, recording days and embryo batches. Supplementary Figure S1 shows normalized distance travelled for each compound and concentration across the eight consecutive 15 min intervals, plotted as median with IQR because the data were right-skewed in several conditions. The corresponding descriptive statistics, including N, mean, SD, median, Q1, Q3 and IQR for each compound, concentration and time interval, are provided in Supplementary Dataset S5. Repeated-measures GEE analysis of ln(normalized distance + 1) confirmed significant concentration, time and concentration × time effects for all compounds, supporting compound-specific and time-dependent modulation of locomotor activity across the 120 min exposure period.

During the initial 0–15 min window, the strongest suppression was already evident for the longer-chain compounds (Table 4). At 5 µM, median BLA was reduced to 37.6% of control for pentylone, 30.8% for dipentylone, 12.9% for dihexylone and 8.5% for diheptylone. By contrast, methylone and butylone produced weaker early effects at the same concentration, with median activities of 88.3% and 82.5% of control, respectively. Dimethylone and dibutylone showed intermediate early inhibition, reaching 62.4% and 53.4% of the control at 5 µM.

The clearest inhibitory phase occurred during the intermediate 45–60 min window (Table 4). At this time point, all compounds showed reduced median activity at 5 µM, with values ranging from 11.2% of control for dipentylone and 12.4% for dihexylone to 42.1% for pentylone. Several compounds also showed marked effects at lower concentrations, particularly methylone, dipentylone, dihexylone and diheptylone. Kruskal–Wallis analyses of representative time windows confirmed significant treatment effects for all compounds at 45–60 min and for most compounds during the early and late windows. Complete Kruskal–Wallis and Dunn–Bonferroni post hoc results for the representative windows are provided in Supplementary Table S4.

During the late 105–120 min window, partial recovery was observed for several analogues, whereas dipentylone, dihexylone and diheptylone retained pronounced locomotor suppression; late-phase descriptive statistics for normalized BLA are provided in Supplementary Table S3. Because the BLA profiles were not consistently monotonic and could not be robustly fitted by conventional nonlinear regression, late-phase locomotor potency was summarized using an interpolated EC_50_-like descriptor. These EC50-like values could be estimated for methylone (4.38 µM), dipentylone (0.145 µM), dihexylone (0.121 µM) and diheptylone (1.32 µM). In contrast, dimethylone, butylone, dibutylone and pentylone did not reach 50% late-phase locomotor inhibition within the tested concentration range (EC_50_-like > 5 µM). Pentylone showed a non-monotonic late-phase pattern, including increased median activity at 500 nM, indicating that late locomotor output did not scale linearly with concentration for all compounds. Analysis of raw immobility parameters over the full 120 min recording supported the hypoactive phenotype observed in BLA. At the highest concentration, most compounds showed increased cumulative immobility duration relative to controls. In several cases, this increase occurred together with a lower frequency of immobility bouts, indicating that locomotor suppression was driven, at least in part, by longer immobility episodes rather than by more frequent short pauses. However, this pattern was not uniform across the series, and some compounds showed increased immobility duration together with unchanged or higher bout frequency. The strongest increases in cumulative immobility duration were observed for the longer-chain analogues, particularly dipentylone, dihexylone and diheptylone (Supplementary Dataset S4).

### 2.5. Cross-Endpoint Comparison of Locomotor and Cardiac Responses

**Figure 4:**
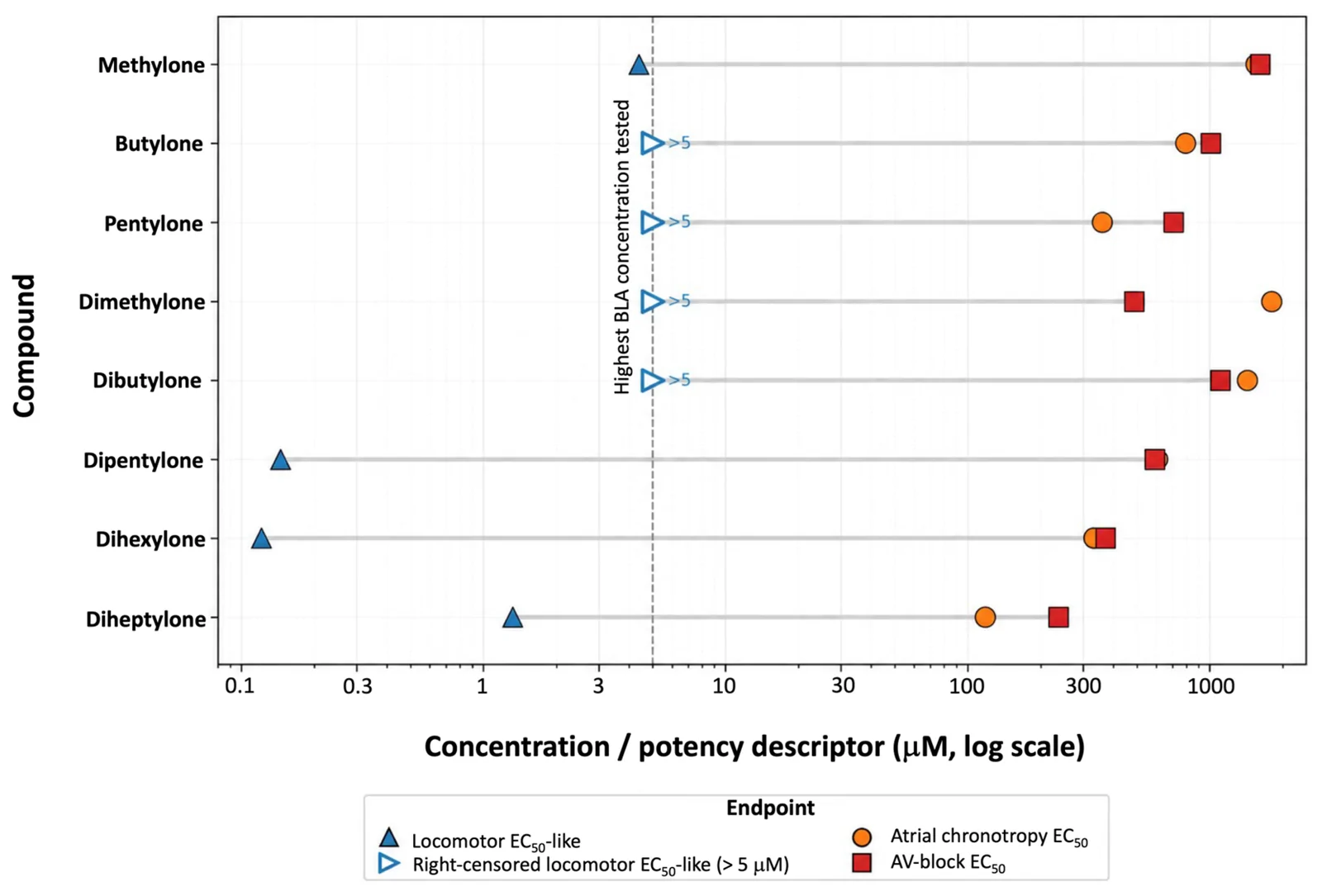
Cross-endpoint comparison of locomotor and cardiac potency descriptors. Interpolated late-phase locomotor EC_50_-like descriptors, constrained 4PL-derived atrial chronotropy midpoint estimates and constrained 4PL-derived AV-block midpoint estimates are displayed on a shared log concentration axis. Right-pointing open triangles positioned at 5 µM indicate compounds for which 50% late-phase locomotor inhibition was not reached within the tested range (>5 µM). Horizontal segments connect locomotor EC_50_-like descriptors with AV-block midpoint estimates within each compound to visualize endpoint separation. This representation is descriptive and does not imply a validated safety index or a shared mechanism between behavioural and cardiac endpoints.

To compare the concentration ranges at which neurofunctional and cardiac effects emerged, late-phase locomotor EC_50_-like descriptors were displayed together with constrained 4PL-derived atrial chronotropy and AV-block midpoint descriptors on a shared log concentration axis. AV block was considered the primary severe cardiac endpoint. This analysis was used only as a descriptive cross-endpoint visualization and was not interpreted as a safety index or as evidence of a shared mechanism between behavioural and cardiac endpoints. The cross-endpoint relationship is visualized in Figure 4 and summarized in Table 5.

Dipentylone and dihexylone showed submicromolar late-phase locomotor EC_50_-like descriptors, whereas their AV-block midpoint descriptors remained in the high-micromolar range. Thus, the concentration ranges associated with locomotor hypoactivity and severe AV conduction impairment were widely separated for these compounds. By contrast, diheptylone combined sustained locomotor suppression with the lowest AV-block midpoint descriptor in the series, indicating a narrower cross-endpoint separation within the tested concentration range.

For dimethylone, butylone, dibutylone and pentylone, 50% late-phase locomotor inhibition was not reached within the tested concentration range, so locomotor EC_50_-like descriptors were reported as right-censored values (>5 µM). Notably, dimethylone displayed prominent AV conduction liability while showing limited late-phase locomotor inhibition at concentrations up to 5 µM, supporting the conclusion that cardiac and neurofunctional endpoints do not necessarily scale in parallel across the series.

## 3. Discussion

### 3.1. Alkyl-Chain Extension Is Associated with Increased Cardiac Liability

The present cardiac dataset reveals a coherent SAR across methylenedioxy cathinones. The most robust pattern was the progressive increase in cardiac liability with increasing alkyl substitution, particularly in the monoalkyl series from methylone to butylone and pentylone, and in the longer dialkyl analogues culminating in dihexylone and diheptylone. This trend was especially evident for atrial chronotropy, where longer-chain compounds consistently produced stronger negative chronotropic effects.

This finding is consistent with the broader synthetic cathinone literature, which shows that alpha-alkyl chain length strongly influences biological potency, pharmacokinetics and in vivo effects [3,9]. SAR analyses indicate that side-chain extension can increase DAT inhibition potency over a defined range and can also modify locomotor activity, although additional chain extension may produce discrepancies between in vitro potency and in vivo behavioural output, possibly through changes in metabolism, tissue distribution, or nonspecific toxicity [3,9,22,23]. Indeed, elongation of the α-carbon chain of different synthetic cathinones has been proposed to increase lipophilicity, thereby enhancing membrane permeability and contributing to greater cytotoxicity [22,23], even in compounds that are not the most potent DAT inhibitors [22]. The present zebrafish data extend this logic to cardiac function by showing that chain extension also modifies atrial rhythmicity and AV conduction liability.

### 3.2. Cardiac Phenotype Severity Shifts from Rate Depression to Conduction Failure

A second relevant observation is that the toxic phenotype became qualitatively more severe in the longer and more hydrophobic analogues. Shorter-chain compounds mainly reduced beat frequency, whereas the more potent compounds increasingly disrupted atrial-ventricular coupling. This suggests that structural modifications do not simply scale a single endpoint but may shift the system from negative chronotropy toward broader electrophysiological dysfunction.

The distinction between atrial rate inhibition and AV block is important because these endpoints are related but not equivalent. Negative chronotropy may reflect impaired pacemaker activity, altered autonomic-like modulation, general cardiodepression or effects on repolarization. In contrast, AV block reflects failure of atrial impulses to propagate effectively to the ventricle, a more severe conduction phenotype. Previous zebrafish work has shown that bradycardia and conduction abnormalities can be detected as separate functional signatures, and that cardiac rhythm and conduction liabilities may be uncoupled across closely related cathinone analogues [16,21,24].

In the present series, diheptylone sits at the extreme end of this toxicity continuum. Its marked effects at 250 µM and among surviving embryos at 500 µM, together with partial mortality at 500 µM and complete lethality at 1000 µM, indicate that the upper concentration range should not be interpreted as a routine segment of a functional concentration-response curve. Rather, it represents a transition toward overt embryotoxicity that constrains the upper end of the evaluable response range. This is relevant for SAR interpretation because further alkyl extension appears to shift the response from functional cardiotoxicity toward overt embryotoxicity within the tested concentration range. As discussed above, this observation is consistent with previous studies showing that α-carbon side-chain elongation is associated with increased in vitro toxicity in human aortic endothelial (HAE) cells as well as in NGF-differentiated PC12 cells [22,23].

The partial mortality observed for diheptylone at 500 µM and dihexylone at 1000 µM also introduces a potential survivorship bias. Functional cardiac measurements at these concentrations necessarily reflect only embryos that remained viable at the time of recording. If the most sensitive embryos died before measurement, atrial-rate and AV-conduction effects calculated from surviving embryos may underestimate the total toxic impact of these treatments at the group level. Accordingly, these concentrations should be interpreted as combined functional cardiotoxic/embryotoxic conditions rather than as purely electrophysiological endpoints.

### 3.3. Terminal Amine Substitution Shapes Endpoint-Specific Profiles

The comparison between N-methyl and N,N-dimethyl analogues suggests that terminal amine substitution does not produce a uniform increase in all cardiac endpoints. For atrial chronotropy, pentylone was more potent than dipentylone, and methylone was more potent than dimethylone, based on EC_50_ rankings. However, dimethylone displayed prominent AV conduction liability, producing high AV block at 500 and 1000 µM and yielding a lower AV block EC_50_ than methylone. Thus, terminal amine dimethylation may alter the balance between rate and conduction effects rather than simply increasing or decreasing overall toxicity.

This endpoint-specific behaviour is consistent with the general principle that cathinone SAR depends on the interaction between multiple structural features, including aromatic substitution, side-chain length, and amine substitution [3,9]. Previous studies demonstrate that changes in the terminal amine can modify transporter potency, DAT/SERT selectivity and behavioural efficacy [8,9]. The present results suggest that cardiac endpoints may be similarly sensitive to terminal amine substitution, but not necessarily in a way that parallels monoamine transporter SAR.

### 3.4. Locomotor Profiling Reveals Neurofunctional Effects That Only Partially Track Cardiac Liability

Time-resolved locomotor profiling added an important neurofunctional dimension to the cardiac SAR. Rather than producing sustained hyperlocomotion, the tested methylenedioxy cathinones predominantly reduced locomotor activity in zebrafish eleutheroembryos. This pattern was especially evident during the intermediate phase of exposure and at the highest concentration tested. As discussed in previous zebrafish studies with cathinones, hypoactivity in this assay should not be interpreted as absence of stimulant pharmacology, but rather as a functional neurobehavioural phenotype that may reflect behavioural shutdown, motor impairment, excessive monoaminergic stimulation or broader systemic toxicity at higher exposure levels [21,24].

Notably, the compounds showing the greatest late-phase locomotor potency were not necessarily those with the highest relative cardiac liability. Dipentylone and dihexylone produced submicromolar locomotor EC_50_-like descriptors, whereas AV block required concentrations in the high-micromolar range, indicating a marked separation between neurofunctional and severe cardiac effects within the tested range. By contrast, diheptylone combined sustained locomotor suppression with the lowest AV-block midpoint descriptor in the series, indicating a comparatively narrower separation between endpoints. Methylone also showed a broad separation, although its locomotor potency was substantially lower. Overall, these compound-specific profiles indicate that increasing alkyl bulk can enhance both neurofunctional and cardiac effects, but does not determine a uniform relationship between the two domains.

### 3.5. Implications for Zebrafish-Based NAMs and Structure-Based Prioritization

The present findings support the value of zebrafish embryos as a multiparametric NAM for comparative hazard prioritization of emerging synthetic cathinones. Zebrafish cardiac physiology retains features relevant to drug-induced rhythm and conduction toxicity, including sensitivity to repolarization-disrupting compounds [16,17]. The model also allows rapid parallel assessment of lethality, atrial rhythm, ventricular rhythm and AV coupling, providing functional resolution beyond mortality or gross morphology alone [15,20,21].

For forensic and public health purposes, these data suggest that methylenedioxy cathinones should not be treated as a homogeneous toxicological class. Instead, small structural changes can shift the dominant phenotype and alter the margin between functional cardiac effects and overt embryotoxicity. The pronounced liability of diheptylone indicates that extension to longer alkyl chains may represent a structural alert for increased acute cardiac hazard in this zebrafish assay.

Several limitations should be acknowledged. First, functional modelling was based on a limited number of non-zero concentrations, so EC_50_ and Hill slope values should be interpreted as comparative descriptors. Second, diheptylone at 1000 µM produced lethality and could not be incorporated into functional endpoint modelling. Third, zebrafish exposure concentrations cannot be directly translated to human plasma concentrations because uptake, distribution, protein binding and metabolism differ across species and experimental systems. Fourth, locomotor EC_50_-like values were derived from only three non-zero concentrations and should therefore be interpreted as assay-specific comparative estimates rather than definitive behavioural potency constants. Finally, the mechanistic basis of the observed cardiac effects remains unresolved; targeted follow-up studies with cardiac ion-channel assays, calcium imaging, or electrophysiological approaches would be needed to distinguish hERG/KCNH2, sodium-channel, calcium-channel, mitochondrial, or membrane-disruptive mechanisms.

## 4. Materials and Methods

### 4.1. Zebrafish Maintenance and Embryo Collection

Adult wild-type short-fin zebrafish (*Danio rerio*) used as breeders were maintained in a recirculating aquatic system (Aquaneering Inc., San Diego, CA, USA) under standard laboratory conditions (28 ± 1 °C; 12 h:12 h light/dark photoperiod). Breeding groups were placed in spawning tanks the day before each experiment. Spawning was induced at lights-on, and fertilized eggs were collected within 30 min.

Embryos were inspected under a stereomicroscope (Nikon SMZ1500, Champigny-sur-Marne, France), and only morphologically normal embryos were selected. Embryos were maintained in embryo water (Milli-Q water supplemented with Instant Ocean salts (Aquarium Systems, Sarrebourg, France) and CaSO_4_·2H_2_O; pH 6.5–7.0; conductivity 750–900 µS/cm), in 48-well microplates (one embryo per well; 1 mL per well) at 28 ± 1 °C, until the appropriate developmental stage. Cardiac assays were performed in 3 days post-fertilization (dpf) embryos, whereas locomotor assays were designed for 5 dpf eleutheroembryos. All procedures were approved by the Institutional Animal Care and Use Committees at CID-CSIC and conducted under local governmental authorization (agreement number 11336).

### 4.2. Compounds and Exposure Solutions

The compound panel consisted of methylenedioxy cathinones differing in alpha-alkyl chain length and terminal amine substitution: methylone, butylone, pentylone, dimethylone, dibutylone, dipentylone, dihexylone and diheptylone. The cathinone derivatives were synthesized in racemic form [25] and characterized as described in the Supplementary Information. Stock solutions of 10 mM were freshly prepared in Milli-Q water on the same day of each experiment, and working solutions were prepared by diluting in embryo water. The pH of the solutions was adjusted, when necessary, with 1 M NaHCO_3_ into an alkaline range suitable for the methylenedioxy cathinones. Final exposure-solution pH was measured after dilution in embryo water in two independent experimental preparations and ranged from 8.0 to 8.5 across compounds; compound-specific values are provided in Supplementary Table S5.

### 4.3. Cardiac Functional Assessment

Cardiac function was assessed after acute exposure using the same overall zebrafish imaging workflow as in the previous pyrrolidine-containing cathinone study [21]. In concentration-response experiments, 3 dpf embryos were exposed for 2 h to nominal concentrations of 250, 500 and 1000 µM, together with matched controls. Embryos lacking a detectable heartbeat at the time of recording were considered dead and excluded from functional cardiac endpoint analysis because atrial and ventricular beat frequencies could not be quantified. Mortality/exclusion counts are reported in Table 1 and detailed in Supplementary Dataset S6. After exposure, embryos were immediately processed for cardiac recordings. 

To reduce movement during imaging, embryos were briefly immobilized with tricaine methanesulfonate (MS-222) at 0.08 mg/mL and embedded in methylcellulose on depression slides. Cardiac activity was recorded laterally at 28 ± 1 °C using a stereomicroscope (SMZ1500, Nikon, Champigny sur Marne, France) coupled to a high-speed camera (ace U acA1440-220um, Basler AG, Ahrensburg, Germany). Atrial and ventricular beat frequencies were quantified from pixel-intensity fluctuations over time using DanioScope v1.0 software (Noldus IT, Wageningen, The Netherlands).

The atrioventricular ratio (R) was calculated as atrial rate divided by ventricular rate (A/V). AV conduction impairment was expressed as percentage AV block using: AV block (%) = 100 × (1 − 1/R), which maps normal 1:1 conduction to 0% and complete ventricular failure toward 100%. Negative chronotropy was expressed as the percent inhibition of atrial rate relative to the matched control: chronotropy (%) = 100 × (1 − A_treated/A_control). Chronotropy values were clipped to the 0–100% range for bounded concentration-response modelling.

### 4.4. Locomotor Activity

Basal locomotor activity (BLA) was assessed as the neurofunctional endpoint using the same general approach as the previous zebrafish cathinone study [21]. Briefly, 5 dpf zebrafish eleutheroembryos were exposed continuously to each compound at 50 nM, 500 nM or 5 µM, together with matched controls. Locomotor behaviour was recorded over 120 min using a DanioVision automated video-tracking system controlled by EthoVision XT 11 software (Noldus IT, Wageningen, The Netherlands). Temperature was maintained at 28 ± 1 °C using a Temperature Control Unit, and recordings were performed under constant dark conditions, without programmed light/dark transitions or light-evoked stimulus periods.

Activity was quantified in consecutive 15 min intervals from 0–15 to 105–120 min. For each compound, concentration and time interval, BLA was expressed as a percentage of the time-matched control median. The predefined reporting windows were 0–15, 45–60 and 105–120 min, representing early, intermediate and late exposure phases. Thirty-six eleutheroembryos were analyzed per condition. Cumulative immobility duration and the frequency of immobility bouts were also extracted over the full 120 min assay, and the relationship between both variables was used to interpret whether hypoactivity reflected more frequent pauses or longer immobility episodes.

### 4.5. Concentration-Response Modelling and Statistics

Cardiac concentration-response relationships were modelled in GraphPad Prism version 11.0.1 (GraphPad Software, Boston, MA, USA) using four-parameter logistic (4PL) functions constrained between 0 and 100%. Constrained 4PL-derived midpoint estimates and Hill slope values were estimated for atrial chronotropy and AV block. Parameter uncertainty was reported as 95% confidence intervals when estimated by the nonlinear regression procedure. Because only three nominal non-zero exposure levels were available, these constrained midpoint and Hill slope estimates should be interpreted only as comparative, SAR-oriented descriptors rather than definitive pharmacodynamic constants. Statistical comparisons of cardiac endpoints against matched controls were performed separately from concentration-response modelling, as summarized in Supplementary Table S2 and reported in full in Supplementary Dataset S2.

Because BLA responses were time-dependent and did not consistently follow monotonic sigmoidal concentration-response relationships, conventional 4PL EC_50_ values were not fitted for locomotor activity. Instead, an assay-specific late-phase locomotor EC_50_-like descriptor was calculated from the 105–120 min interval by log-linear interpolation between the two concentrations bracketing 50% residual activity. When 50% inhibition was not reached within the tested range, the EC_50_-like value was treated as right-censored (>5 µM). To compare the concentration ranges at which locomotor and cardiac endpoints emerged, late-phase locomotor EC_50_-like descriptors, constrained atrial chronotropy midpoint descriptors and constrained AV-block midpoint descriptors were displayed on a shared log concentration axis. This cross-endpoint comparison was used only as an assay-specific descriptive visualization and was not interpreted as a safety index or as evidence of a shared mechanism between behavioural and cardiac endpoints.

For cardiac endpoints, statistical comparisons were performed using an endpoint-specific approach summarized in Supplementary Table S2, with the complete output provided in Supplementary Dataset S2. For atrial rate inhibition, normality was assessed using the Shapiro–Wilk test and homogeneity of variances using Levene’s test. Depending on the outcome of these assumption checks, groups were analyzed using one-way ANOVA followed by Dunnett’s post hoc test, Welch ANOVA followed by Dunnett T3 post hoc comparisons, or Kruskal–Wallis followed by Dunn–Bonferroni comparisons versus matched controls. For AV block, Kruskal–Wallis tests followed by Dunn–Bonferroni post hoc comparisons were used because this endpoint is bounded between 0 and 100%, is zero-inflated, and frequently included constant control or low-effect groups.

For locomotor endpoints, normality was assessed using the Shapiro–Wilk test, and non-parametric Kruskal–Wallis tests followed by Dunn–Bonferroni post hoc comparisons were used for the predefined representative windows. These analyses were retained as window-specific comparisons and were not interpreted as a model of the complete longitudinal dataset. In addition, the complete 0–120 min locomotor time course was analyzed separately for each compound using generalized estimating equations (GEE). Normalized distance travelled was transformed as ln(normalized distance + 1) and analyzed with concentration, time interval and concentration × time interaction as model effects. Embryo identity was used as the subject variable to account for repeated observations from the same eleutheroembryo, and time interval was specified as the within-subject factor. A Gaussian distribution with identity link, AR(1) working correlation structure and robust covariance estimator were used. Type III Wald χ^2^ tests were used to evaluate concentration, time and concentration × time effects. Complete GEE results and time-resolved normalized distance-travelled descriptive statistics are provided in Supplementary Dataset S5. Statistical significance was set at *p* < 0.05.

## 5. Conclusions

Increased alkyl bulk within methylenedioxy cathinones is associated with progressively greater cardiac hazard in zebrafish embryos. In the monoalkyl series, methylone < butylone < pentylone for negative chronotropy, whereas longer dialkyl analogues, especially diheptylone, shift the phenotype toward severe AV conduction failure and overt embryotoxicity. Locomotor profiling revealed predominantly hypoactive neurofunctional phenotypes, with dipentylone and dihexylone showing the strongest late-phase locomotor potency. Integration of both domains indicates that neurofunctional and cardiac endpoints are partially dissociated: locomotor alterations generally occur at much lower concentrations than AV block, but endpoint separation varies across analogues. These results support multiparametric zebrafish profiling as a useful SAR-oriented approach for prioritizing emerging methylenedioxy cathinones with increased cardiac liability.

## Figures and Tables

**Table 1:** Mortality/exclusions during the cardiac assay.

Compound	250 µM	500 µM	1000 µM
Methylone	0/16	0/15	0/16
Butylone	0/16	0/16	0/16
Pentylone	0/16	0/16	0/16
Dimethylone	0/16	0/16	0/16
Dibutylone	0/14	0/16	0/16
Dipentylone	0/16	0/15	0/16
Dihexylone	0/15	0/16	5/16
Diheptylone	0/15	3/15	16/16

**Table 2:** Atrial chronotropy parameters derived from 4PL concentration-response modelling.

Compound	EC_50_ (µM)	95% CI (EC_50_)	Hill Slope	95% CI (Hill)
Diheptylone	118.3	45.6–168.5	0.56	0.30–0.83
Dihexylone	332.5	296.6–366.6	0.88	0.72–1.04
Pentylone	359.6	332.4–386.3	1.08	0.94–1.21
Dipentylone	608.5	551.8–676.8	0.90	0.74–1.06
Butylone	792.2	750.6–839.7	1.42	1.28–1.57
Dibutylone	1428.0	1247.0–1712.0	0.99	0.82–1.16
Methylone	1552.0	1342.0–1902.0	1.42	1.15–1.72
Dimethylone	1804.0	1537.0–2233.0	0.81	0.69–0.94

Note: EC_50_ and Hill slope values were estimated using a 4PL model constrained between 0 and 100%. Diheptylone at 1000 µM was excluded from fitting due to complete lethality. Hill slope values are reported as positive magnitudes because the endpoint is expressed as percent inhibition.

**Table 3:** AV block parameters derived from 4PL concentration-response modelling.

Compound	EC_50_ (µM)	95% CI (EC_50_)	Hill Slope	95% CI (Hill)
Diheptylone	237.3	N.E.	19.15	N.E.
Dihexylone	370.8	301.0–455.6	3.35	1.75–6.65
Dimethylone	488.8	N.E.	35.42	N.E.
Dipentylone	593.7	458.1–794.2	3.13	N.E.
Pentylone	707.1	706.2–708.0	7.94	7.91–7.97
Butylone	1007.0	N.E.	34.92	N.E.
Dibutylone	1107.0	846.9–2330.0	2.06	0.87–5.24
Methylone	1610.0	1301.0–2285.0	2.30	1.50–3.92

Note: AV block was defined as loss of 1:1 atrial-ventricular coupling. Model estimates should be interpreted cautiously because only three nominal exposure levels were available, and one diheptylone condition was removed due to lethality. N.E., not estimated by the nonlinear regression procedure. Hill slope values are reported as positive magnitudes because the endpoint is expressed as percent AV block.

**Table 4:** Summary of locomotor activity in representative time windows. Values are medians expressed as a percentage of the time-matched control median and are shown for 50 nM/500 nM/5 µM. Because BLA responses were time-dependent and did not consistently follow monotonic sigmoidal concentration-response relationships, conventional nonlinear-regression EC_50_ values were not fitted for locomotor activity. Locomotor EC_50_-like values were instead estimated from the 105–120 min interval by log-linear interpolation when 50% residual activity was bracketed by the tested concentrations. When 50% inhibition was not reached at 5 µM, values are reported as >5 µM.

Compound	0–15 min	45–60 min	105–120 min	Locomotor EC_50_-like (µM)
Methylone	94.9/105.7/88.3	25.9/24.5/23.3	106.4/78.7/48.2	4.38
Butylone	103.1/107.6/82.5	88.8/45.4/22.8	65.8/57.3/50.4	>5
Pentylone	101.0/93.1/37.6	47.1/67.0/42.1	93.8/221.4/66.2	>5
Dimethylone	104.0/97.8/62.4	69.7/20.0/26.5	89.0/64.5/50.1	>5
Dibutylone	102.0/100.9/53.4	60.8/74.2/28.8	88.9/79.1/54.7	>5
Dipentylone	99.1/73.4/30.8	44.8/27.9/11.2	72.7/23.5/20.7	0.145
Dihexylone	88.9/59.0/12.9	31.8/47.3/12.4	54.3/43.1/11.9	0.121
Diheptylone	106.0/86.0/8.5	55.9/39.0/14.1	70.1/66.9/26.8	1.32

**Table 5:** Cross-endpoint comparison of locomotor and cardiac potency descriptors. Late-phase locomotor EC_50_-like descriptors, constrained 4PL-derived atrial chronotropy midpoint estimates and constrained 4PL-derived AV-block midpoint estimates are shown on a shared concentration scale. Values are presented only as assay-specific comparative descriptors and should not be interpreted as a validated safety index or as evidence of a shared mechanism between locomotor and cardiac endpoints. Locomotor EC_50_-like values > 5 µM indicate that 50% late-phase locomotor inhibition was not reached within the tested concentration range.

Compound	Atrial EC_50_ (µM)	AV Block EC_50_ (µM)	Locomotor EC_50_-like (µM)
Methylone	1552.0	1610.0	4.38
Butylone	792.2	1007.0	>5
Pentylone	359.6	707.1	>5
Dimethylone	1804.0	488.8	>5
Dibutylone	1428.0	1107.0	>5
Dipentylone	608.5	593.7	0.145
Dihexylone	332.5	370.8	0.121
Diheptylone	118.3	237.3	1.32

## Data Availability

The data supporting the findings of this study are available within the manuscript and its Supplementary Materials or will be made available from the corresponding author upon request.

## Supplementary Materials

The following supporting information can be downloaded at https://www.mdpi.com/article/10.3390/ph19081243/s1: Supplementary Methods. Synthesis and characterization of the methylenedioxy cathinones; Table S1. Experimental design, replication, exclusions and recording conditions for the zebrafish cardiac and locomotor assays; Table S2. Statistical approach used for the analysis of cardiac endpoints; Table S3. Late-phase (105–120 min) descriptive statistics for normalized BLA by compound and concentration; Table S4. Complete nonparametric statistics for locomotion at representative windows: Kruskal–Wallis and Dunn’s post hoc tests (Bonferroni-adjusted) versus control; Table S5. Final pH values of exposure solutions after dilution in embryo water. Values correspond to two independent experimental preparations; Figure S1. Time-resolved normalized distance travelled (% control median) during the 120 min basal locomotor activity assay; Dataset S1. Normalized cardiac chamber heart rate; Dataset S2. Statistical analysis of cardiac endpoints; Dataset S3. Median normalized basal locomotor activity and log2 fold-change values; Dataset S4. Raw immobility parameters during the 120 min BLA assay; Dataset S5. Time-resolved normalized distance-travelled descriptive statistics and repeated measures; Dataset S6. Experimental metadata, exclusions and endpoint definitions; Supplementary Video S1. Control 3 dpf zebrafish embryo showing normal 1:1 atrioventricular coupling; Supplementary Video S2. Diheptylone 250 μM showing marked bradycardia with preserved 1:1 atrioventricular coupling; Supplementary Video S3. Diheptylone 250 μM showing 2:1 atrioventricular block; Supplementary Video S4. Diheptylone 500 μM showing severe 2:1 atrioventricular block among surviving embryos; Supplementary Video S5. Dihexylone 1000 μM showing severe 2:1 atrioventricular block among surviving embryos.
