# Supplementary material for: Alkyl-Chain Extension and Terminal Amine Substitution Shape Cardiotoxic Profiles of Methylenedioxy Cathinones in Zebrafish Embryos
Source: Pharmaceuticals (Basel). 2026 Aug 7;19(8):1243. doi: 10.3390/ph19081243 (PMC13516094; doi:10.3390/ph19081243)
Supplement: Supplementary file 1 [file pharmaceuticals-19-01243-s001.zip › pharmaceuticals-4465306-supplementary.pdf]

Supplementary Information

Alkyl-Chain Extension and Terminal Amine Substitution Shape Cardiotoxic Profiles of Methylenedioxy Cathinones in Zebrafish Embryos

Ouwais Aljabasini, Niki Tagkalidou, Martalu D. Pazos, Guillermo García-Díez, Eva Prats, Roger Seco, Xavier Berzosa, Raúl López-Arnau, and Demetrio Raldua

Supplementary Methods

S1. Synthesis and characterization of the methylenedioxy cathinones

S1.1. Synthesis

The synthesis of the different cathinones was performed through three sequential steps, following the procedure described by Meltzer et al. [25]. The overall route of synthesis is shown in the following figure.

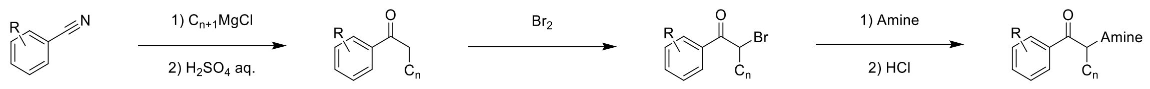

In general, the substituted benzonitrile is reacted with the appropriate Grignard reagent to obtain the corresponding ketone, after an acidic treatment. Afterwards, the ketone is brominated in the α position. Finally, the bromoketone is reacted with the corresponding amine to obtain the desired cathinone as a free base. The hydrochloride salt can be produced by reaction with hydrogen chloride.

S1.2. Characterization

A Varian 400-MR spectrometer has been used to record ^{1}H-NMR and ^{13}C-NMR spectra. Chemical shifts are reported in part per million (ppm) on the δ scale. Coupling constants are reported in Hertz (Hz).

The absence of relevant organic impurity signals in the ^{1}H-NMR and ^{13}C-NMR spectra, together with the purification procedure used, supports an estimated purity >95% for the synthesized compounds. Significant inorganic contamination was considered unlikely because the compounds were dissolved in low-polarity organic solvent prior to acidification with HCl, whereas inorganic impurities would be expected to remain insoluble and to be removed by filtration before crystallization.

Methylone

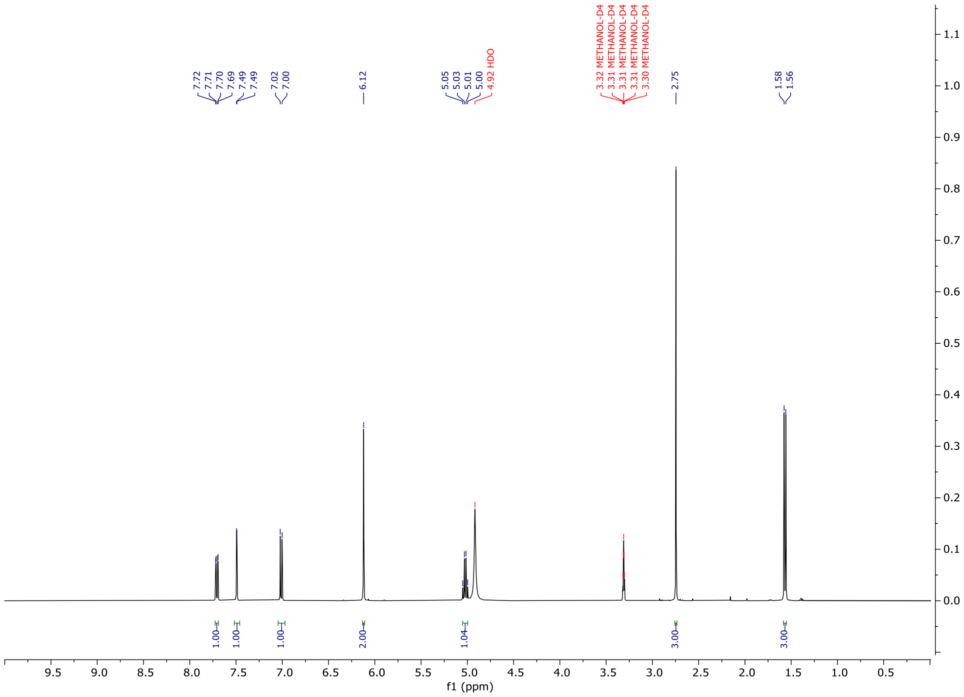

^{1}H NMR (400 MHz, CD_3OD) δ 7.71 (dd, *J* = 8.3, 1.8 Hz, 1H), 7.49 (d, *J* = 1.8 Hz, 1H), 7.01 (d, *J* = 8.3 Hz, 1H), 6.12 (s, 2H), 5.02 (q, *J* = 7.2 Hz, 1H), 2.75 (s, 3H), 1.57 (d, *J* = 7.2 Hz, 3H).

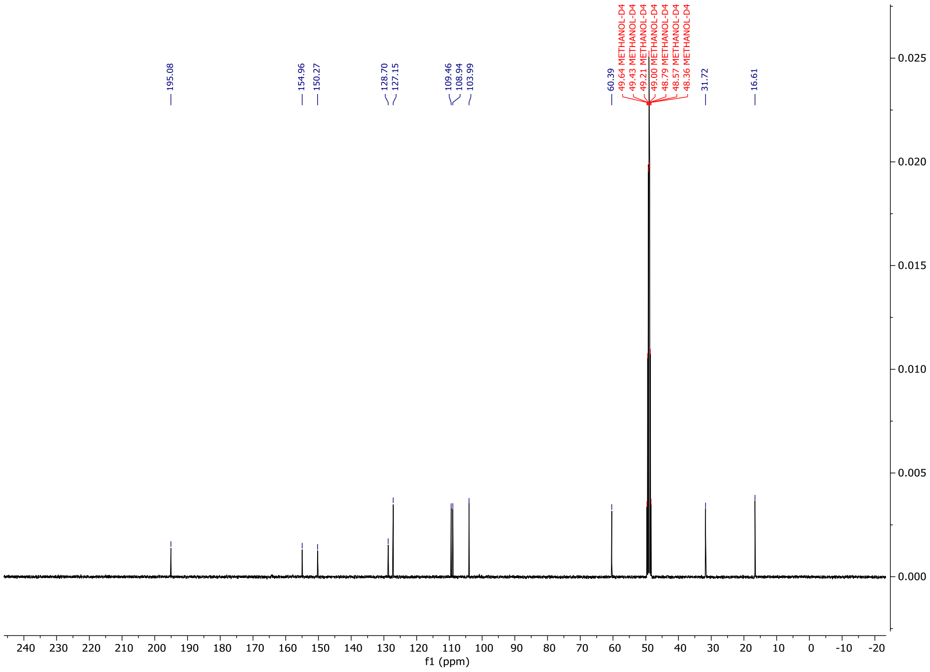

^{13}C NMR (101 MHz, CD_3OD) δ 195.08, 154.96, 150.27, 128.70, 127.15, 109.46, 108.94, 103.99, 60.39, 31.72, 16.61.

Dimethylone

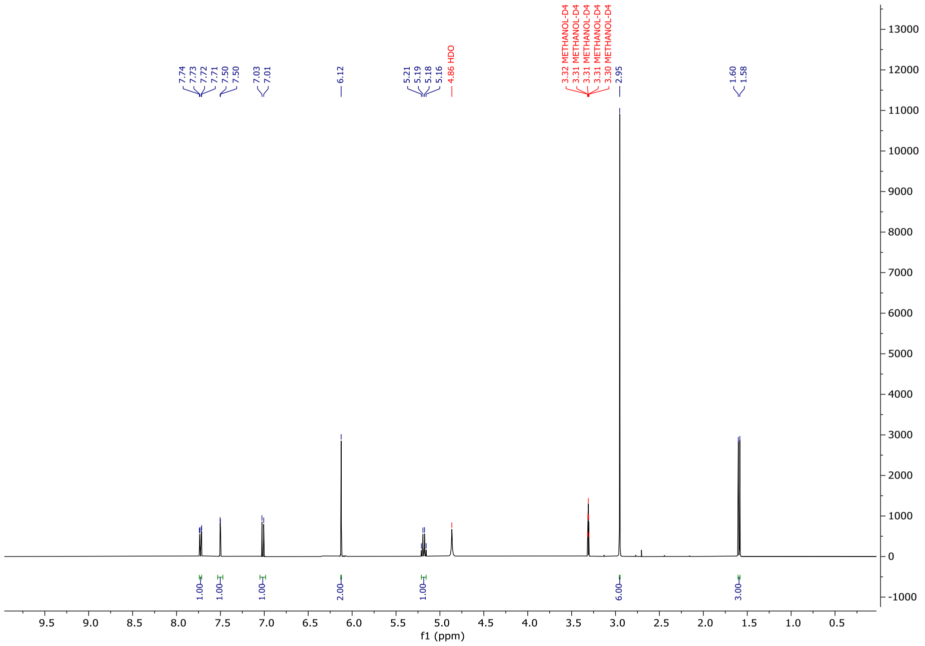

^{1}H NMR (400 MHz, CD_3OD) δ 7.73 (dd, *J* = 8.3, 1.8 Hz, 1H), 7.50 (d, *J* = 1.5 Hz, 1H), 7.02 (d, *J* = 8.2 Hz, 1H), 6.12 (s, 2H), 5.18 (q, *J* = 7.2 Hz, 1H), 2.95 (s, 6H), 1.59 (d, *J* = 7.2 Hz, 3H).

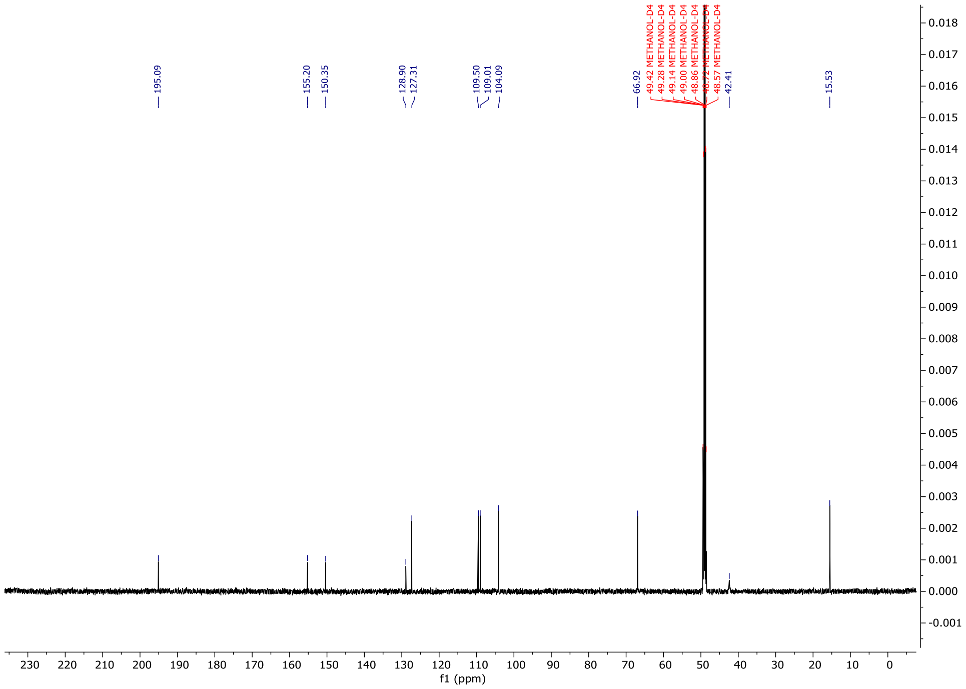

^{13}C NMR (151 MHz, CD_3OD) δ 195.09, 155.20, 150.35, 128.90, 127.31, 109.50, 109.01, 104.09, 66.92, 42.41, 15.53.

Butylone

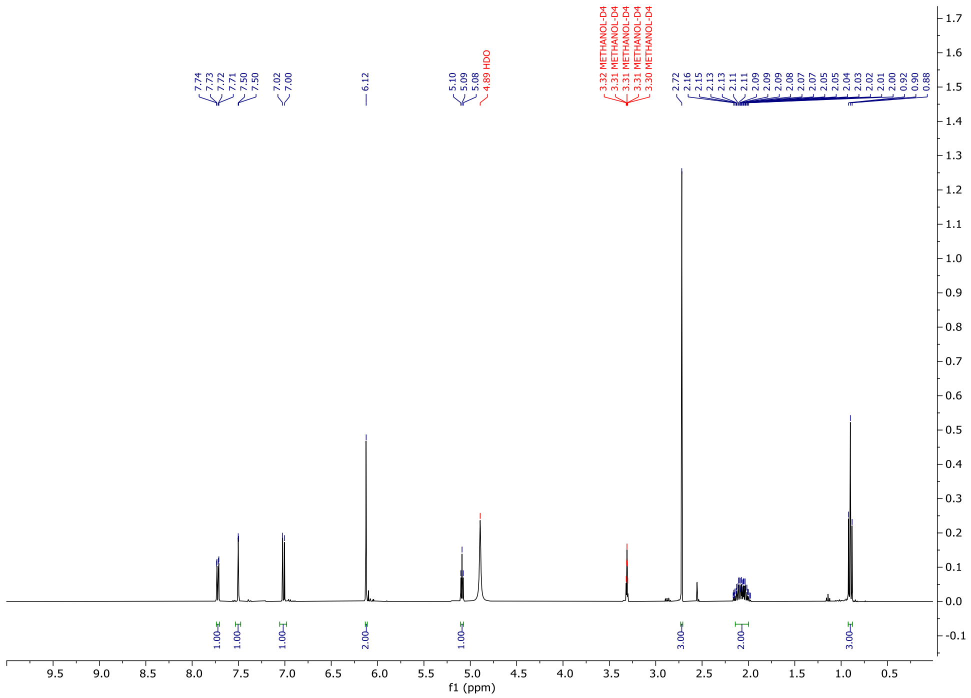

^{1}H NMR (400 MHz, CD_3OD) δ 7.72 (dd, J = 8.3, 1.8 Hz, 1H), 7.50 (d, J = 1.8 Hz, 1H), 7.01 (d, J = 8.3 Hz, 1H), 6.12 (s, 2H), 5.09 (t, J = 5.2 Hz, 1H), 2.72 (s, 3H), 2.14 – 2.00 (m, 2H), 0.90 (t, J = 7.6 Hz, 3H).

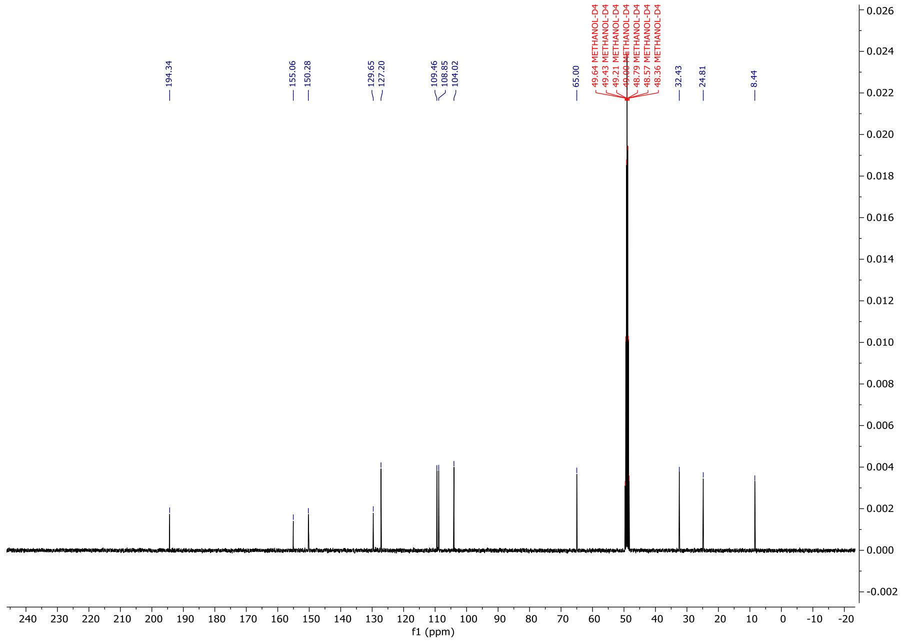

^{13}C NMR (101 MHz, CD_3OD) δ 194.34, 155.06, 150.28, 129.65, 127.20, 109.46, 108.85, 104.02, 65.00, 32.43, 24.81, 8.44.

Dibutylone

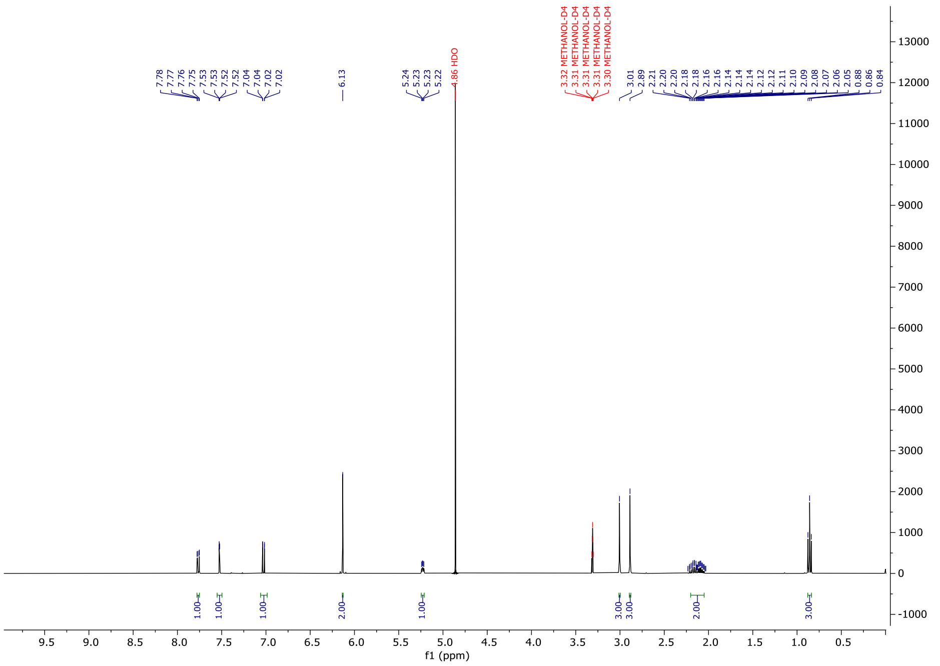

^{1}H NMR (400 MHz, CD_3OD) δ 7.77 (dd, *J* = 8.3, 1.8 Hz, 1H), 7.53 (dd, *J* = 1.9, 0.4 Hz, 1H), 7.03 (dd, *J* = 8.2, 0.4 Hz, 1H), 6.13 (s, 2H), 5.23 (dd, *J* = 6.5, 4.0 Hz, 1H), 4.86 (s, 5H), 3.01 (s, 3H), 2.89 (s, 3H), 2.20 – 2.05 (m, 2H), 0.86 (t, *J* = 7.5 Hz, 3H).

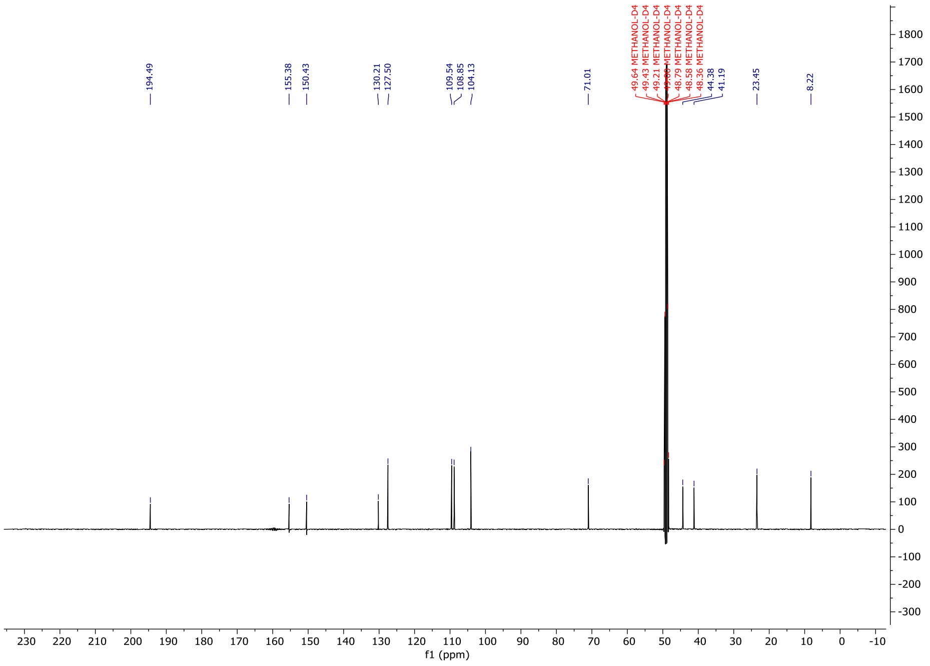

^{13}C NMR (100 MHz, CD_3OD) δ 194.49, 155.38, 150.43, 130.21, 127.50, 109.54, 108.85, 104.13, 71.01, 44.38, 41.19, 23.45, 8.22.

Pentylone

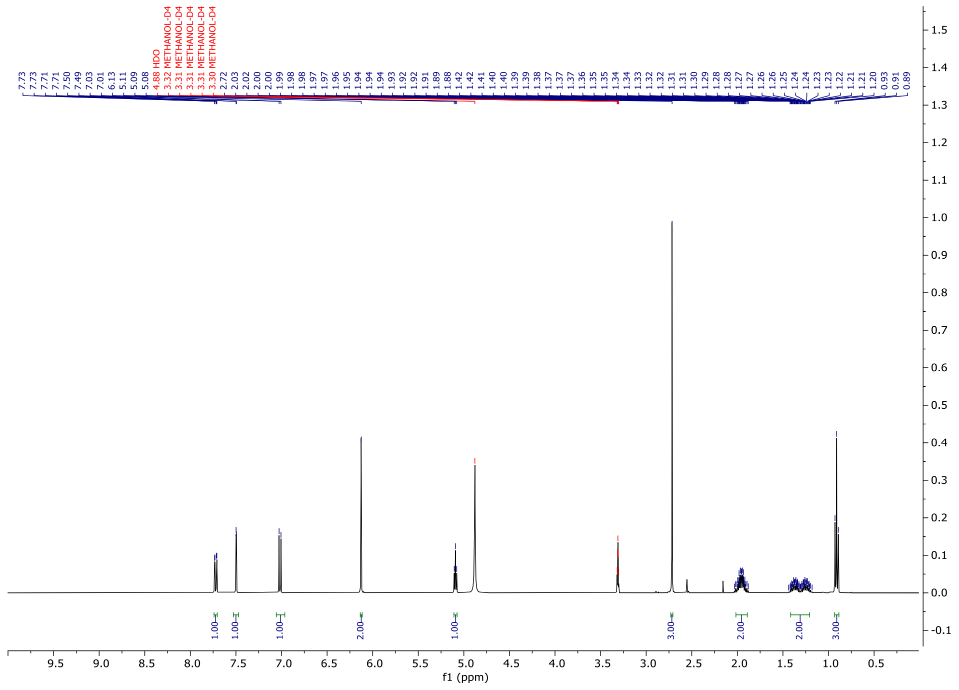

^{1}H NMR (400 MHz, CD_3OD) δ 7.72 (dd, *J* = 8.3, 1.8 Hz, 1H), 7.50 (d, *J* = 1.8 Hz, 1H), 7.02 (d, *J* = 8.2 Hz, 1H), 6.13 (s, 2H), 5.09 (t, *J* = 5.5 Hz, 1H), 2.72 (s, 3H), 2.02 – 1.89 (m, 2H), 1.41 – 1.21 (m, 2H), 0.91 (t, *J* = 7.3 Hz, 3H).

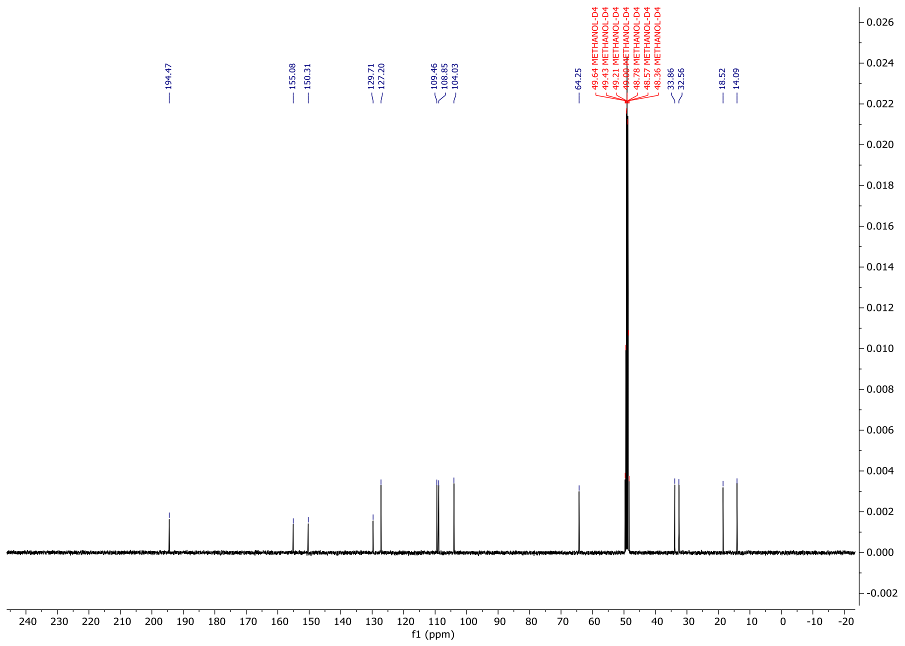

^{13}C NMR (101 MHz, CD_3OD) δ 194.47, 155.08, 150.31, 129.71, 127.20, 109.46, 108.85, 104.03, 64.25, 33.86, 32.56, 18.52, 14.09.

Dipentylone

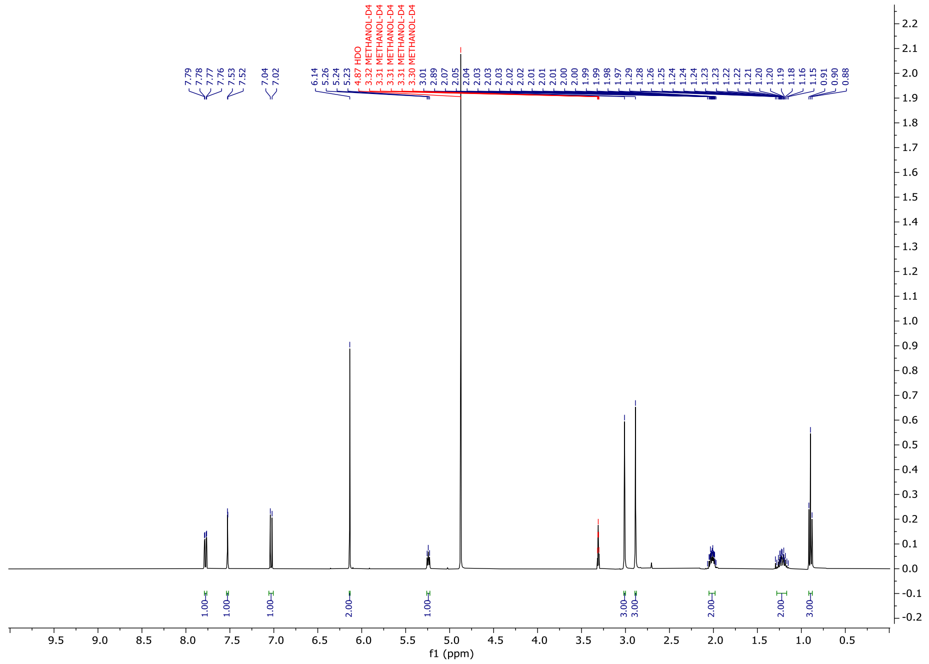

^{1}H NMR (400 MHz, CD_3OD) δ 7.78 (dd, *J* = 8.3, 1.8 Hz, 1H), 7.52 (d, *J* = 1.8 Hz, 1H), 7.03 (d, *J* = 8.2 Hz, 1H), 6.14 (s, 2H), 5.26 – 5.22 (m, 1H), 3.01 (s, 3H), 2.89 (s, 3H), 2.05 – 1.98 (m, 2H), 1.28 – 1.17 (m, 2H), 0.90 (t, *J* = 7.3 Hz, 3H).

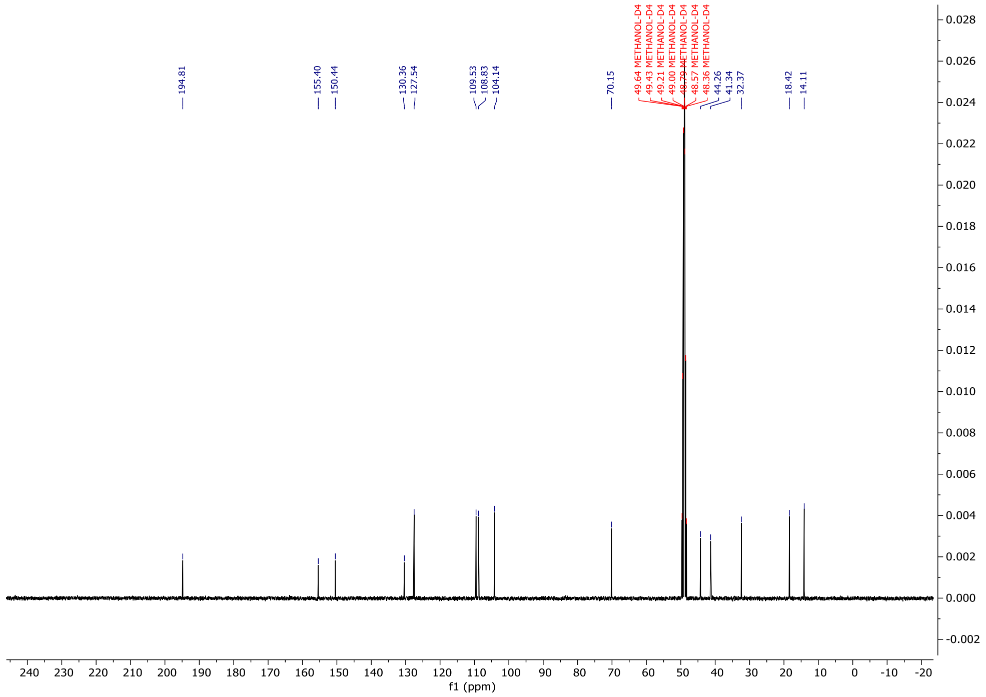

^{13}C NMR (101 MHz, CD_3OD) δ 194.81, 155.40, 150.44, 130.36, 127.54, 109.53, 108.83, 104.14, 70.15, 44.26, 41.34, 32.37, 18.42, 14.11.

Dihexylone

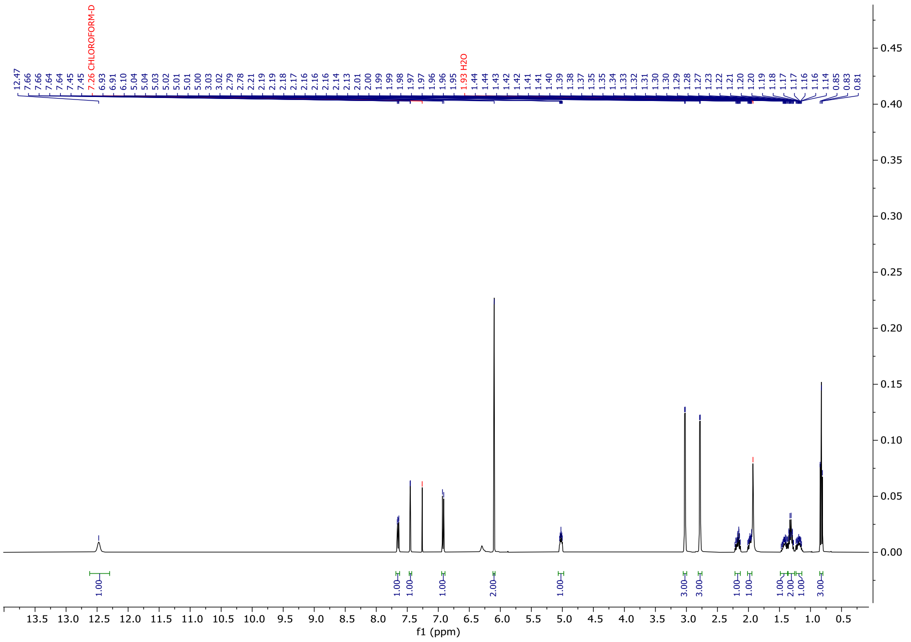

^1H NMR (400 MHz, $CDCl_3$) δ 12.47 (s, 1H), 7.65 (dd, *J* = 8.3, 1.8 Hz, 1H), 7.45 (d, *J* = 1.6 Hz, 1H), 6.92 (d, *J* = 8.2 Hz, 1H), 6.10 (s, 2H), 5.02 (ddd, *J* = 8.8, 5.2, 3.2 Hz, 1H), 3.02 (d, *J* = 4.9 Hz, 3H), 2.78 (d, *J* = 4.9 Hz, 3H), 2.22 – 2.13 (m, 1H), 1.98 (ddd, *J* = 14.4, 8.5, 3.5 Hz, 1H), 1.49 – 1.37 (m, 1H), 1.36 – 1.26 (m, 2H), 1.18 (ddt, *J* = 15.6, 6.8, 5.0 Hz, 1H), 0.83 (t, *J* = 7.4 Hz, 3H).

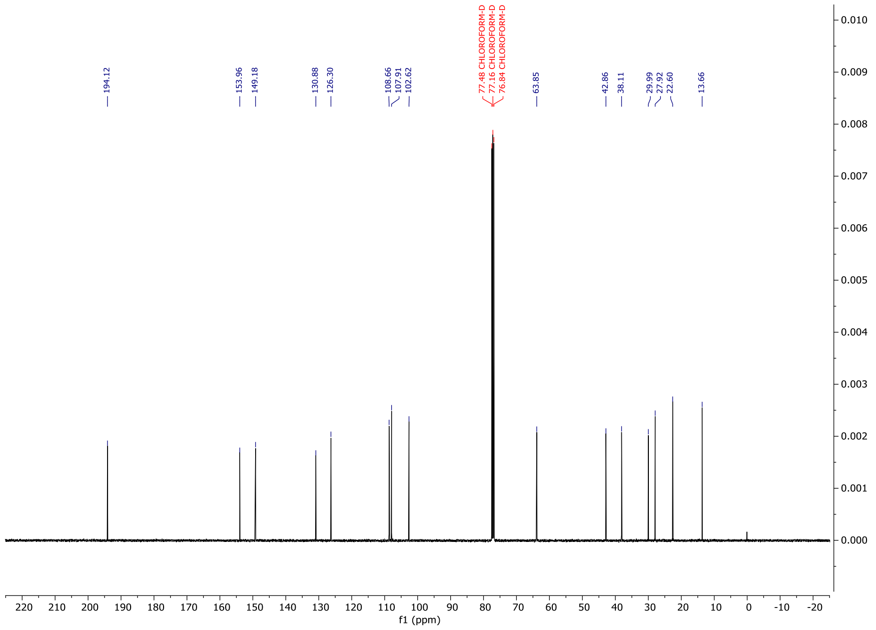

^{13}C NMR (101 MHz, $CDCl_3$) δ 194.12, 153.96, 149.18, 130.88, 126.30, 108.66, 107.91, 102.62, 63.85, 42.86, 38.11, 29.99, 27.92, 22.60, 13.66.

Diheptylone

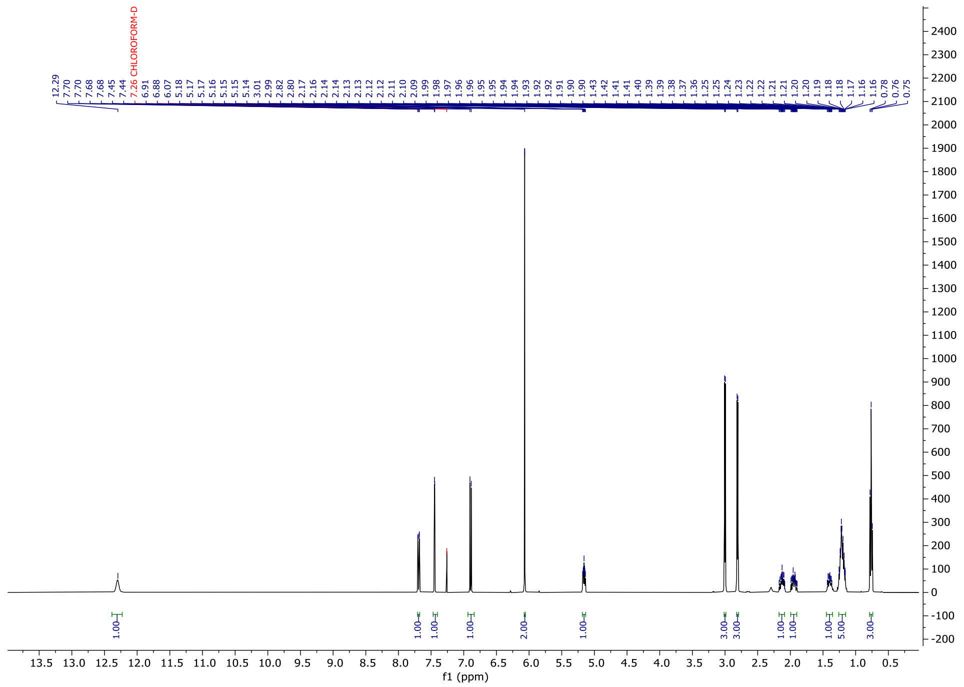

^1H NMR (400 MHz, $CDCl_3$) δ 12.29 (s, 1H), 7.69 (dd, *J* = 8.3, 1.8 Hz, 1H), 7.45 (d, *J* = 1.9 Hz, 1H), 6.89 (d, *J* = 8.3 Hz, 1H), 6.07 (s, 2H), 5.16 (ddd, *J* = 8.8, 5.1, 3.6 Hz, 1H), 3.00 (d, *J* = 5.0 Hz, 3H), 2.81 (d, *J* = 4.9 Hz, 3H), 2.17 – 2.09 (m, 1H), 1.95 (dtd, *J* = 11.3, 9.0, 5.7 Hz, 1H), 1.45 – 1.35 (m, 1H), 1.26 – 1.15 (m, 5H), 0.76 (t, *J* = 7.0 Hz, 3H).

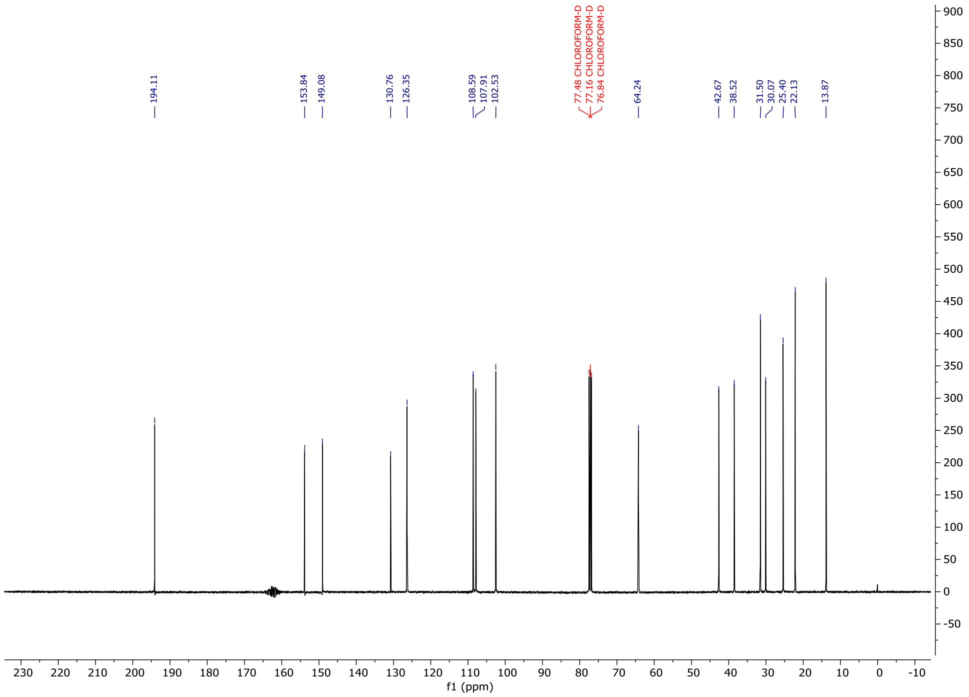

^{13}C NMR (100 MHz, $CDCl_3$) δ 194.11, 153.84, 149.08, 130.76, 126.35, 108.59, 107.91, 102.53, 64.24, 42.67, 38.52, 31.50, 30.07, 25.40, 22.13, 13.87.

Supplementary Figures

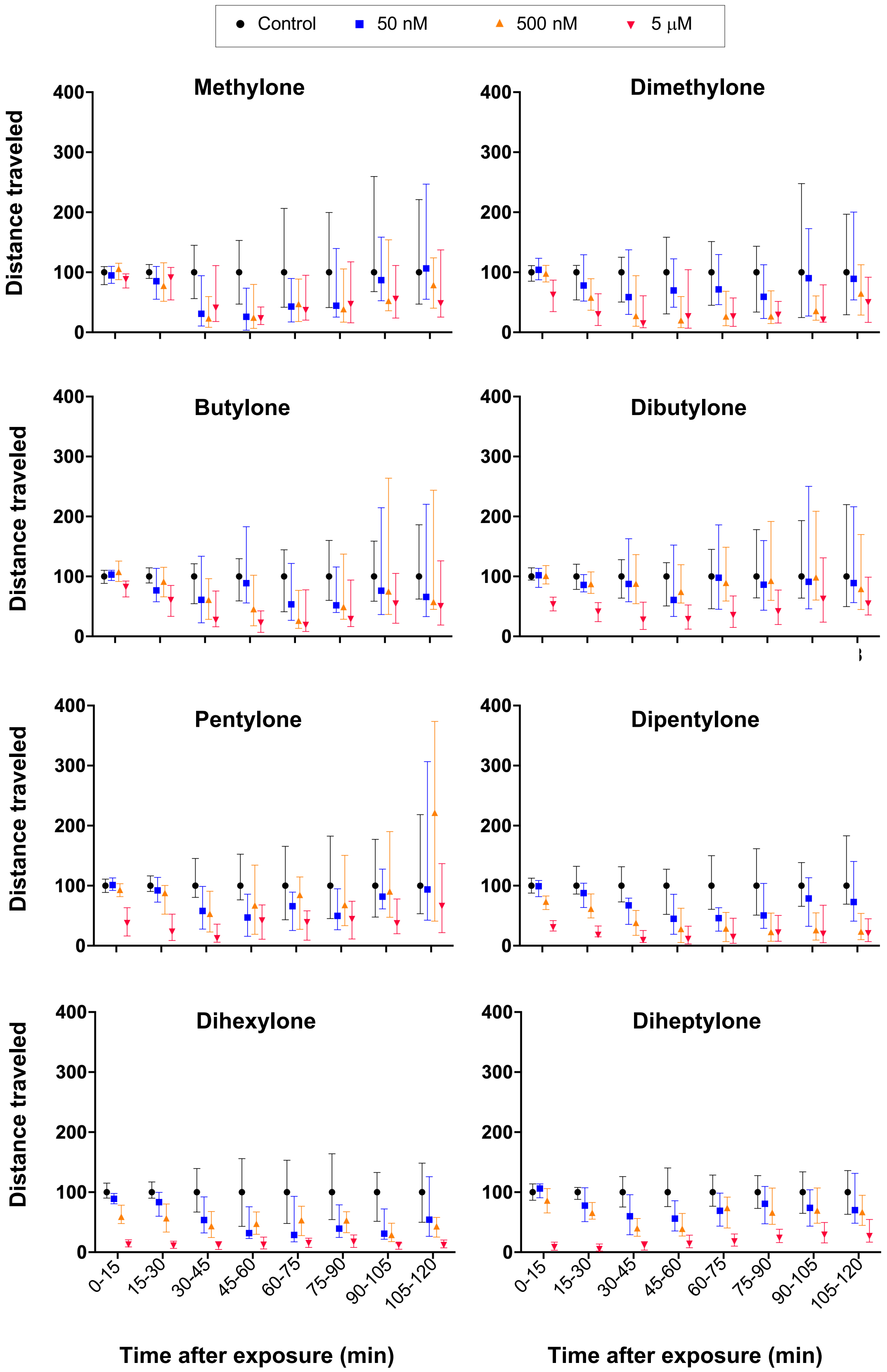

Supplementary Figure S1. Time-resolved normalized distance travelled (% control median) during the 120 min basal locomotor activity assay. Distance travelled was quantified in consecutive 15 min intervals in 5 dpf zebrafish eleutheroembryos exposed to each methylenedioxy cathinone at 50 nM, 500 nM or 5 µM, together with matched controls. Each panel corresponds to one compound. Points represent median normalized distance travelled and vertical bars indicate the interquartile range (IQR). Points were horizontally offset within each time interval to improve visualization of overlapping concentration groups. Descriptive statistics, including N, mean, SD, median, Q1, Q3 and IQR for each compound, concentration and time interval, together with repeated-measures GEE results, are provided in Supplementary Dataset S5.

Supplementary Tables

Table S1. Experimental design, replication, exclusions and recording conditions for the zebrafish cardiac and locomotor assays.

Assay	Stage	Exposure	Experimental unit	Replication	Recording conditions	Main endpoints	Detailed data
Cardiac assay	3 dpf embryos	120 min; 250, 500, 1000 µM	Individual embryo	14–16 embryos/group	28 ± 1 °C; MS-222: 0.08 mg/mL; high-speed cardiac imaging	Atrial rate, ventricular rate, AV block, mortality	Datasets S1, S2 and S6
Locomotor assay	5 dpf eleutheroembryos	120 min; 50 nM, 500 nM, 5 µM	Individual eleutheroembryo	3 batches; 12/batch; 36/group	28 ± 1 °C; constant dark conditions; DanioVision	Distance/BLA, log2 fold-change, immobility	Datasets S3, S4, S5 and S6

Table S2. Statistical approach used for the analysis of cardiac endpoints.

Endpoint	Assumption checks	Statistical approach	Post hoc comparisons	Complete output
Atrial rate inhibition	Normality was assessed using the Shapiro–Wilk test. Homogeneity of variances was assessed using Levene's test.	When assumptions were met, one-way ANOVA was used. When variance homogeneity was not met, Welch ANOVA was used. When normality was not met, Kruskal–Wallis test was used.	Dunnett's test after one-way ANOVA; Dunnett T3 after Welch ANOVA; Dunn–Bonferroni after Kruskal–Wallis. Comparisons were performed versus the matched control group.	Dataset S2
AV block	The distributional structure was inspected. Because AV block is bounded between 0 and 100%, zero-inflated, and frequently included constant control or low-effect groups, parametric assumptions were not considered appropriate for this endpoint.	Kruskal–Wallis test.	Dunn–Bonferroni post hoc comparisons versus the matched control group.	Dataset S2

Table S3. Late-phase (105–120 min) descriptive statistics for normalized BLA by compound and concentration.

Compound	Condition	Dose (µM)	Median [IQR]	n
**Methylone**	Control	0	100.00 [46.26-247.68]	36
	50 nM	0.05	106.37 [54.11 – 254.08]	36
	500 nM	0.5	78.73 [34.89 – 131.64]	36
	5 µM	5	48.24 [23.98 – 138.02]	36
**Butylone**	Control	0	100 [60.48 – 186.35]	36
	50 nM	0.05	65.79 [33.00 – 243.67]	36
	500 nM	0.5	57.32 [43.12 – 279.28]	36
	5 µM	5	50.39 [16.61 – 130.59]	36
**Pentylone**	Control	0	100 [50.50 – 220.72]	36
	50 nM	0.05	93.81 [42.47 – 318.39]	36
	500 nM	0.5	221.42 [40.08 – 377.21]	36
	5 µM	5	66.16 [21.69 – 136.83]	36
**Dimethylone**	Control	0	100 [28.65 – 207.35]	36
	50 nM	0.05	89.01 [50.97 – 210.33]	36
	500 nM	0.5	64.53 [28.58 – 114.46]	36
	5 µM	5	50.12 [16.26 – 94.19]	36
**Dibutylone**	Control	0	100 [49.70 – 223.47]	36
	50 nM	0.05	88.90 [56.22 – 216.37]	36
	500 nM	0.5	79.07 [41.36 – 176.74]	36
	5 µM	5	54.67 [33.26 – 100.19]	36
**Dipentylone**	Control	0	100 [67.85 – 194.05]	36
	50 nM	0.05	72.73 [39.25 – 152.04]	36
	500 nM	0.5	23.53 [10.04 – 62.47]	36
	5 µM	5	20.68 [6.25 – 37.81]	36
**Dihexylone**	Control	0	100 [48.67 – 167.82]	36
	50 nM	0.05	54.25 [26.29 – 126.53]	36
	500 nM	0.5	43.14 [23.59 – 59.14]	36

	5 µM	5	11.87 [7.19 – 20.50]	36
**Diheptylone**	Control	0	100 [62.06 – 136.33]	36
	50 nM	0.05	70.06 [47.51 – 132.69]	36
	500 nM	0.5	66.90 [41.93 – 97.59]	36
	5 µM	5	26.76 [16.15 – 63.12]	36

Table S4. Complete nonparametric statistics for locomotion at representative windows: Kruskal–Wallis and Dunn's post hoc tests (Bonferroni-adjusted) versus control.

Compound	Window (min)	*H*	*p*	Dunn *p* (50 nM)	Dunn *p* (500 nM)	Dunn *p* (5 µM)
**Methylone**	0-15	7.10	0.069	1.000	1.000	0.278
	45-60	21.26	<0.001	<0.001	<0.001	<0.001
	105-120	7.70	0.053	1.000	1.000	0.275
**Butylone**	0-15	28.43	<0.001	1.000	1.000	0.002
	45-60	27.49	<0.001	1.000	0.158	<0.001
	105-120	9.39	0.025	0.982	1.000	0.015
**Pentylone**	0-15	53.27	<0.001	1.000	0.912	<0.001
	45-60	24.46	<0.001	0.003	0.091	<0.001
	105-120	9.52	0.023	1.000	1.000	0.221
**Dimethylone**	0-15	34.14	<0.001	1.000	1.000	<0.001
	45-60	18.75	<0.001	1.000	0.003	0.018
	105-120	8.95	0.030	1.000	0.729	0.087
**Dibutylone**	0-15	58.44	<0.001	1.000	1.000	<0.001
	45-60	24.46	<0.001	1.000	1.000	<0.001
	105-120	9.08	0.028	1.000	1.000	0.112
**Dipentylone**	0-15	91.29	<0.001	1.000	<0.001	<0.001
	45-60	44.66	<0.001	0.020	<0.001	<0.001
	105-120	49.43	<0.001	0.573	<0.001	<0.001
**Dihexylone**	0-15	96.53	<0.001	0.383	<0.001	<0.001
	45-60	47.43	<0.001	0.013	0.069	<0.001
	105-120	61.81	<0.001	0.156	0.002	<0.001
**Diheptylone**	0-15	83.98	<0.001	1.000	0.413	<0.001
	45-60	61.62	<0.001	0.026	<0.001	<0.001
	105-120	27.08	<0.001	1.000	0.738	<0.001

Table S5. Final pH values of exposure solutions after dilution in embryo water. Values correspond to two independent experimental preparations.

Compound	Experiment 1	Experiment 2
Methylone	8.0	8.2
Butylone	8.1	8.2
Pentylone	8.2	8.1
Dimethylone	8.3	8.1
Dibutylone	8.2	8.3
Dipentylone	8.3	8.5
Dihexylone	8.5	8.3
Diheptylone	8.4	8.3

Supplementary Dataset

Dataset S1. **Normalized cardiac chamber heart rate**. Normalized atrial (A) and ventricular (V) heart-rate values in 3 days post-fertilization zebrafish embryos under control conditions and after exposure to the eight tested compounds at 250, 500 or 1000 µM. Values are expressed relative to the corresponding control group and were used to calculate atrial rate inhibition, A/V ratio and AV block.

Dataset S2. Statistical analysis of cardiac endpoints. Complete statistical output for the cardiac endpoint analyses. The dataset includes group-level descriptive statistics, assumption checks, statistical test selection, global test statistics and adjusted post hoc comparisons versus the corresponding control group for atrial rate inhibition and AV block. For atrial rate inhibition, Shapiro–Wilk normality tests and Levene's tests for homogeneity of variances were used to guide the choice of one-way ANOVA, Welch ANOVA or Kruskal–Wallis analysis, followed by the appropriate post hoc comparison versus matched controls. For AV block, non-parametric analysis was used because the endpoint is bounded between 0 and 100%, zero-inflated and frequently includes constant control or low-effect groups.

Dataset S3. Median normalized basal locomotor activity and log2 fold-change values. Median normalized basal locomotor activity values and corresponding log2 fold-change values for each compound, concentration and 15-min recording interval. Normalized activity values are expressed as percentage of the time-matched control median. Log2 fold-change values were calculated relative to the corresponding control group and used for heatmap visualization in Figure 3.

Dataset S4. **Raw immobility parameters during the 120 min BLA assay**. Non-normalized cumulative immobility duration, frequency of immobility bouts and mean immobility bout duration measured over the full 0–120 min recording period for each compound and concentration. Cumulative immobility duration is expressed in seconds, frequency corresponds to the number of immobility bouts during the recording period, and mean immobility bout duration was calculated as cumulative immobility duration divided by bout frequency. Values are shown for individual 5 dpf zebrafish eleutheroembryos under control conditions and after exposure to 50 nM, 500 nM or 5 µM of each compound.

Dataset S5. **Time-resolved normalized distance-travelled descriptive statistics and repeated-measures GEE analysis.** Descriptive statistics and repeated-measures analysis of distance travelled during the 120 min basal locomotor activity assay after normalization to the corresponding time-matched control median. The dataset includes GEE results for each compound, using ln(normalized distance + 1) as the response variable, concentration, time interval and concentration × time interaction as model effects, embryo identity as the subject variable, time interval as the within-subject factor, an AR(1) working correlation structure and robust covariance estimator. The dataset also provides time-resolved descriptive statistics for normalized distance travelled, including N, mean, SD, median, Q1, Q3 and IQR for each compound, concentration and 15 min recording interval. These data support Supplementary Figure S1.

Dataset S6. **Experimental metadata, exclusions and endpoint definitions.** Assay-level metadata supporting reproducibility of the cardiac and locomotor assays. The dataset includes a summary of experimental design, developmental stage, exposure duration, concentration ranges, experimental unit, number of independent experimental runs, embryos per run,

recording hardware/software, temperature and illumination conditions. It also reports cardiac mortality/exclusion counts by compound and concentration, locomotor replication structure by compound and concentration, and definitions/formulas for the main cardiac, locomotor and integrated endpoints used in the study.

Supplementary Videos

Supplementary Video S1. Control 3 dpf zebrafish embryo showing normal 1:1 atrioventricular coupling. Representative cardiac recording from a control embryo. Atrial and ventricular rates were both 199 beats per minute (bpm), corresponding to 97% of the matched control reference value for both chambers. The atrioventricular ratio was 1:1, indicating normal atrioventricular coupling.

Supplementary Video S2. Diheptylone 250 µM showing marked bradycardia with preserved 1:1 atrioventricular coupling. Representative cardiac recording from a 3 dpf zebrafish embryo exposed to diheptylone at 250 µM. Atrial and ventricular rates were both 56 bpm, corresponding to 29% of the matched control value for both chambers. The atrioventricular ratio was 1:1, indicating severe negative chronotropy without overt atrioventricular conduction block in this embryo.

Supplementary Video S3. Diheptylone 250 µM showing 2:1 atrioventricular block. Representative cardiac recording from a second 3 dpf zebrafish embryo exposed to diheptylone at 250 µM. The atrial rate was 100 bpm and the ventricular rate was 50 bpm, corresponding to 45% and 22% of the matched control value, respectively. The atrioventricular ratio was 2:1, indicating atrioventricular conduction impairment.

Supplementary Video S4. Diheptylone 500 µM showing severe 2:1 atrioventricular block among surviving embryos. Representative cardiac recording from a surviving 3 dpf zebrafish embryo exposed to diheptylone at 500 µM. The atrial rate was 70 bpm and the ventricular rate was 35 bpm, corresponding to 31% and 16% of the matched control value, respectively. The atrioventricular ratio was 2:1, illustrating severe conduction impairment at a concentration associated with partial mortality.

Supplementary Video S5. Dihexylone 1000 µM showing severe 2:1 atrioventricular block among surviving embryos. Representative cardiac recording from a surviving 3 dpf zebrafish embryo exposed to dihexylone at 1000 µM. The atrial rate was 47 bpm and the ventricular rate was 24 bpm, corresponding to 22% and 11% of the matched control value, respectively. The atrioventricular ratio was approximately 2:1, illustrating severe atrioventricular conduction impairment at a concentration associated with partial mortality.
